# Molecular marker identification, antioxidant, antinociceptive, and anti-inflammatory responsiveness of malonic acid capped silver nanoparticle

**DOI:** 10.3389/fphar.2023.1319613

**Published:** 2024-01-31

**Authors:** Tehrim Fatima, Hina Abrar, Noor Jahan, Sana Shamim, Nazia Ahmed, Asma Basharat Ali, Irshad Begum, Waqas Ahmed

**Affiliations:** ^1^ Department of Pharmacology, Dow College of Pharmacy, Faculty of Pharmaceutical Sciences, Dow University of Health Sciences, Karachi, Pakistan; ^2^ Department of Pharmaceutical Chemistry, Dow College of Pharmacy, Faculty of Pharmaceutical Sciences, Dow University of Health Sciences, Karachi, Pakistan; ^3^ Dow Research Institute of Biotechnology and Biosciences, Dow University of Health Sciences, Karachi, Pakistan; ^4^ Department of Anatomy, Jinnah Medical and Dental College, Karachi, Pakistan; ^5^ Department of Chemistry, University of Karachi, Karachi, Pakistan; ^6^ School of Public Health, Dow University of Health Sciences, Karachi, Pakistan

**Keywords:** silver nanoparticles, anti-inflammatory potential, antinociceptive agent, antioxidant efficacy, malonic acid, sodium borohydride

## Abstract

Nano-sized silver has drawn a great deal of attention in the field of health sciences owing to its remarkable therapeutic applications. Interestingly, the method applied to synthesize nanoparticles and the choice of reagents considerably influence their therapeutic potential and toxicities. Current research has explored the toxicity, anti-inflammatory, antinociceptive, and antioxidant responses of the malonic acid-capped silver nanoparticles (MA-AgNPs (C) by using sodium borohydride as a reducing agent at low temperatures by employing both *in vitro* and *in vivo* approaches. Furthermore, it has highlighted the synergistic effect of these novel compounds with conventional anti-inflammatory therapeutic agents. Acute and sub-acute toxicity analysis performed following OECD guidelines showed that the studied MA-AgNPs (C) are safer, and prominent toxic signs have not been detected at the highest studied dose of 2,000 mg/kg. Cytotoxicity evaluation through brine shrimp lethality revealed 20% lethality at the highest concentration of 169.8 μg/mL. Significantly, positive anti-inflammatory and analgesic responses alone as well as synergism with the standard were identified through *in vitro* as well as *in vivo* methods which were more potent at a lower dose (200 mg/kg). Notably synergistic outcomes were more pronounced than individual ones, indicating their prominent effect as a feasible drug delivery system. IL-6 and TNF-α assessment in excised paw tissue through RTPCR technique further supported their anti-inflammatory potential. DPPH assay revealed eminent *in vitro* antioxidant activity which was further corroborated by *in vivo* antioxidant assessment through evaluation of SOD in excised paw tissue.

## 1 Introduction

Inflammation is a defense mechanism by which the immune system identifies and eliminates harmful and foreign stimuli, allowing the body to recover (Michels da Silva et al., 2019). The regularly occurring protective inflammatory process offers defense against any infection, facilitates healing, and preserves the tissue’s natural functioning (Fritsch and Abreu, 2019; Zhang et al., 2019). The primary problem is not the likelihood of occurrence but rather the failure of repair (Pahwa et al., 2021). Restorative procedures and maintenance of homeostasis depend heavily on inflammation. Nonetheless, disruption and augmentation of this defensive host immune response may become the origin of several pathological ailments (Atsumi et al., 2014; Hirano, 2021). Steroidal and non-steroidal anti-inflammatory medications recommended by clinical practitioners in current practice to manage mild to chronic inflammatory conditions are linked with several dose-dependent adverse reactions (Hersh et al., 2011; Schjerning et al., 2020). Therefore, regardless of their existence, the discovery of safer, more acceptable, and more efficient therapeutic anti-inflammatory treatments is in dire need.

Silver metal has been valued as a remarkable medicinal agent since antiquity. It has been used as a remarkable antibacterial agent for at least six thousand years. Consequently, as antibiotics began to be used in clinical settings in the 1940s, silver’s medical applications began to wane. However, due to antibiotic abuse, antibacterial drug resistance has now emerged as a significant health risk. As a result, silver is once again becoming popular in the 21st century, especially with the advent of nanotechnology (Barillo and Marx, 2014; Medici et al., 2019).

Nanotechnology has made significant strides in this era. Due to its outstanding *in vitro* and *in vivo* medicinal benefits, it is generating a lot of fascination in the field of medicine. There is a substantial amount of interest in nanotechnology research. Moreover, nanometals are strong contenders for use in a variety of biological and industrial applications because of their high surface-to-volume ratio and distinctive physical, chemical, and biological properties (Sharma et al., 2009). They are now recognized as a crucial research tool because of their quickly developing therapeutic capabilities, in particular, gold and silver are increasingly used for drug delivery and medical diagnostics (Yang et al., 2012). Additionally, nano-sized silver has drawn a huge interest, making it a potential agent for the medical sector due to its antibacterial, anticancer, anti-inflammatory, anticoagulant, and anti-aging activities (Castro-Aceituno et al., 2016).

The morphology and surface charge of AgNPs are strongly influenced by the selection of certain reagents (reducing or capping agents), pH, and temperature, which subsequently modify their medicinal potential. The present research aims to improve the efficiency and lessen the undesirable side effects of anti-inflammatory and analgesic drugs by examining the therapeutic potential of malonic acid-capped silver nanoparticles (MA-AgNPs (C)) synthesized using the cold method at a temperature of 4°C. Specifically, the study will focus on assessing their *in vitro* and *in vivo* anti-inflammatory, antinociceptive, and antioxidant properties. The therapeutic responses were assessed both individually as well as in synergism with FDA-approved drugs for the analysis of their synergistic response. Shamim S. et al. have already synthesized and characterized these malonic acid-capped AgNPs, and their antibacterial potency has been investigated (Begum et al., 2021). The distinctiveness of this research is the application of the particular MA-AgNPs (C), whose toxicity profiling, cytotoxicity evaluation, *in vivo* and *in vitro* anti-inflammatory, antinociceptive, and antioxidant properties explored here have not been reported previously. Since the uniqueness of the synthetic approach makes them distinct from other AgNPs, their therapeutic efficacy ought to be specific.

## 2 Materials and methods

### 2.1 Materials

Malonic acid-capped silver nanoparticles, Diclofenac Sodium (Na) was provided as a gift from Abott Pharmaceutical (Pvt) Ltd., Pakistan. Acetaminophen (GlaxoSmithKlein, Pvt Ltd., Pakistan) and vincristine sulphate injectable (Pharmedic Laboratories (Pvt) Ltd., Pakistan) were procured for the study.

### 2.2 Reagents

All the chemicals employed were of analytical grade. Normal saline (0.9%), distilled water, chloroform, egg albumin powder, carrageenan, acetic acid 100%, triazole, isopropanol 70%, 75% ethanol, nuclease free water, 10X reaction buffer (containing MgCl_2_), DNAase kit (Invitrogen Inc.), RevertAid™ First Strand cDNA synthesis kit (Thermo Scientific), Maxima SYBR Green Master Mix 2X (abm, Canada).DPPH, β actin forward and reverse primer, IL-6, and TNF-α forward and reverse primer, RTPCR plates (RNAase free). Pyrex A-grade quality glass wares were used throughout the study.

#### 2.2.1 Synthesis of MA-AgNPs (C)

**SCHEME 1:**
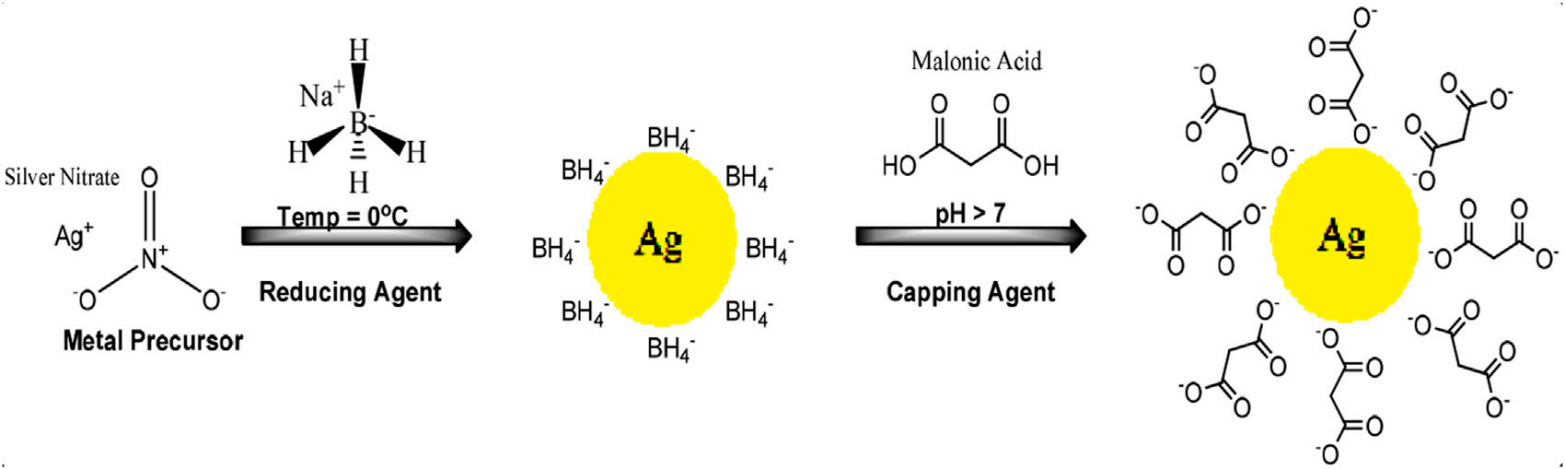
Schematic diagram of the stepwise synthesis mechanism of MA-AgNPs (C).

MA-AgNPs (C) were synthesized by the chemical reduction method as reported by Begum et al. (2021) without any modifications (Scheme 1). In this reported method, silver nitrate was used as the precursor salt that released the Ag^+^ ion utilizing Sodium borohydride (NaBH_4_, 4°C) as a reducing agent, and capped with malonic acid to prevent aggregation, followed by centrifugation to remove impurities. Begum et al., 2021characterized these nanoparticles by UV visible spectroscopy, FT-IR, SEM, TEM, DLS, and zeta potential (Begum et al., 2021).

### 2.3 Animals

Animals were purchased from the animal house of Dow University of Health Sciences (DUHS). Healthy male and female wistar rats, weighing 180–230 g (9–10 weeks old) and swiss albino mice, weighing 20–30 g (8–12 weeks old) were selected and procured. Animals were kept in cages and provided with unrestricted access to food and tap water and were housed at 27°–30°C and a 12 h light/12 h dark cycle, humidity in the environment was kept between 40%–70%. Ethical Approval was taken from the Ethical Review Board for Animal Research and Ethics Committee of Dow University of Health Sciences (Ref No: AR.IRB-21/DUHS/Approval/2022/44). Furthermore, the proposal was approved by the Institutional Board (IRB) of Dow University of Health Sciences (IRB Reference No: IRB-2698/DUHS/Approval/2022/037).

### 2.4 Toxicity testing

#### 2.4.1 Acute toxicity testing

Acute toxicity testing was carried out following the Organization of Economic Co-operation and Development 423 (OECD) guidelines. Healthy swiss albino adult female mice (non-pregnant), weighing 20–30 g and 8–12 weeks old, were randomly categorized into two groups each containing three mice. Group 1 served as control (Distilled water) while Group 2 was administered with a single oral dose of 2,000 mg/kg of MA-AgNPs (C). Animals’ behavior, toxic sign and symptoms, body weight, and mortality were closely monitored for the next 14 days. In the end, animals were sacrificed, and their organs (liver, kidney, and brain) were isolated and histopathological findings were examined (OECD, 2002; Jonsson et al., 2013; Kifayatullah et al., 2015).

#### 2.4.2 Sub-acute toxicity testing

Following the OECD 407 guidelines, healthy swiss albino mice weighing 20–30 g (aged 8–12 weeks) were randomly divided into 2 Groups comprising of 3 mice, each. Group 1 served as control (Distilled water) while group 2 was orally administered with 1,000 mg/kg of MA-AgNPs (C)/day for 14 days. Intermittently behavioral changes, pharmacotoxic signs and symptoms, and mortality were recorded. At the end of the study timeline, the animals were sacrificed, and their liver, kidney, and brain were isolated, for pathological findings under the microscope (OECD, 2008; Kifayatullah et al., 2015).

#### 2.4.3 Histopathology

H&Estained sections of the liver, kidney, and brain of each group (control, acute, and sub-acute toxicity testing) were examined under the microscope.

#### 2.4.4 Brine shrimp lethality bioassay

*Artemia salina* (Brine shrimp) eggs were hatched in seawater that was prepared by dissolving 38 g/L of sea salt in distilled water followed by filtration, ambient temperature of 29°C and appropriate oxygenation maintained for 48 h to promote hatching. Mature nauplii were counted and transferred to test tubes in such a manner that each test tube contained 10 nauplii. Cytotoxicity of MA-AgNPs (C) was evaluated at 0.84, 4.24, 8.5, 17, 84.9, and 169.8 μg/mL by separately introducing them in test tubes. Distilled water was kept as a negative control to avoid false positive results. Positive control vincristine sulfate’s cytotoxicity was simultaneously assessed at 0.06, 0.125, 0.25, 0.5, 1, 5, and 10 μg/mL. The volume of each test tube was made up to 5 mL. Test tubes were retained for 24 h. Percentage mortality at each concentration was determined by counting the alive nauplii in each test tube and applying the formula, LC_50_ was determined by plotting a regression line.

$$\text{Percentage mortality }\left(\%\right)=\left(\text{number of dead nauplii}\right)/\left(\text{initial number of alive nauplii}\right)\times 100$$

### 2.5 Anti-inflammatory activity

#### 2.5.1 *In vitro* protein denaturation method

Protein denaturation inhibition of MA-AgNPs (C) was analyzed alone and in synergism with standard (Diclofenac Na 1,000 μg/mL) at 425 and 850 μg/mL 1 mL (1 mM) of egg albumin solution was mixed with standard and control (Distilled water), 425 and 850 μg/mL of MA-AgNPs (C), and in combination with standard in separate test tubes. After that, the solutions were incubated for 15 min at 27°C ± 1°C followed by subjecting them to a water bath at 70°C for 10 min. The absorbance of each solution in the test tube was examined separately in a spectrophotometer at a wavelength of 660 nm keeping the vehicle as blank. By applying the following formula, the percentage of protein denaturation inhibition of each sample was calculated:

$$\text{Percentage inhibition }\left(\%\right)=\left(\left(\text{Abs of control }–\text{ Abs of the sample}\right)\right)/\left(\text{Abs of control}\right)\times 100$$

IC_50_, i.e., the concentration that causes 50 percent protein denaturation inhibition was determined by applying regression analysis and plotting the percentage inhibition graph (Chopade et al., 2012; Gogoi et al., 2020).

#### 2.5.2 *In vivo* carrageenan-induced paw edema method

Wistar albino rats (n:30, weight: 180–230 g, age: 9–10 weeks) were divided randomly into six groups of control 1), standard 2), and test (3,4, 5, and 6). Administered with distilled water, Diclofenac Na 50 mg/kg, and—200 and 400 mg/kg doses of MA-AgNPs (C)alone and in synergism with standard respectively. The specified dose of the drug was administered orally to each rat as grouped. The Right paw of each rat was marked, and the base paw initial (V_0_) and final volume (V_f_) were recorded using a plethysmometer (panlab 2014, Harvard apparatus). After 30 minutes of oral drug administration, each rat received 0.1 mL of 1% carrageenan in the sub-planter area of the designated paw. The paw volume of each rat was then measured at 1, 2, 3, 4, 5, 6, and 24 h post carrageenan administration and percentage paw volume inhibition was calculated by applying the following formula (Winter et al., 1962; Cordaro et al., 2020).

$$\text{Paw edema inhibition }\left(\%\right)=\left(\left[\left(V_{f}\;–\;V_{0}\right)\text{ control }–\;\left(V_{f}\;–\;V_{0}\right)\text{ treated}\right]\right)/\left(\left(V_{f}\;–\;V_{0}\right)\text{ control}\right)\times 100$$

#### 2.5.3 Measurement of tissue inflammatory cytokines

The paw tissue of the rat was dissected at the 5^th^ hour of post-carrageenan administration, frozen in phosphate-buffered saline (PBS), and then stored at −80°C (Karim et al., 2019). Frozen tissue was thawed, and total RNA was extracted with the help of TRIzol or TRI reagent RNA extraction method (Pandey and Chawla, 2021). After RNA isolation its concentration was determined in Nanodrop Flurospectrophotometer (Thermo Scientific). RNA was purified from DNA contaminants using a DNase kit (Invitrogen Inc.). The cDNA of each sample was synthesized by reverse transcribing their RNA using RevertAid First Strand cDNA synthesis kit (Thermo Scientific) which was carried out by first polymerizing RNA followed by their conversion to cDNA through reverse transcriptase enzyme. The gene expressions of IL-6 and TNF α were analyzed through qRT-PCR using specific primers (Primer sequence Table 1). The primers were purchased from ABM Goods, Canada. RT-PCR was performed by using Maxima SYBR Green Master Mix 2X (Thermo Scientific) and their manufacturer’s manual was followed. The amplification of the samples was performed in PCR QuantStudio 7 Flex Thermalcycler (Thermo Scientific). First, samples were denatured at 95°C for 5 min and then cooled at 50°C–60°C to allow the annealing of each primer for 30 s, and finally, extension was performed at 72°C for 1 min, the cycle was then repeated 40 times. The expression levels of IL-6 and TNF α were normalized to the level of the housekeeping gene (β actin) and determined (Aziz et al., 2019; Bakshi et al., 2022).

**TABLE 1:** Primer sequence used for qRT-PCR.

PCR forward (F) primers	Sequence
β-actin	5′-CAG​GGT​GTG​ATG​GTG​GGT​ATG​G-3′
IL-6	5′-GTG​GCT​AAG​GAC​CAA​GAC​CA-3′
TNF-α	5′-AGC​CCT​GGT​ATG​AGC​CCA​TGT​A-3′
SOD	5′-GCA​CAT​TAA​CGC​GCA​GAT​CA-3′

PCR reverse (R) primers	Sequence
β-actin	5′-AGT​TGG​TGA​CAA​TGC​CGT​GTT​C-3′
IL-6	5′-GGT​TTG​CCG​ACT​AGA​CCT​CA-3′
TNF-α	5′-CCG​GAC​TCC​GTG​ATG​TCT​AAG​T-3′
SOD	5′-AGC​CTC​CAG​CAA​CTC​TCC​TT-3′

### 2.6 Antinociceptive activity

#### 2.6.1 *In vivo* tail flick method

The studied animal group was the same as in Section 2.5.2. For the study, the water bath’s temperature was kept between 50°C–52°C. Each animal’s distal 2–3rd of the tail was marked, immersed in hot water, and the base reading (T_0_ **)**, was measured. The defined doses of the test substance, standard (acetaminophen 300 mg/kg), and control (distilled water) were then given orally to all the animals. Following drug delivery, each mouse’s tail flick reaction was monitored at 15, 30, 60, 90, and 120 min and the percentage protection of each group was calculated through a formula (Sewell and Spencer, 1976; D'Amour and Smith, 1941) where T_0_ represents base response, T_t_ as response at maximum time and T_m_ as maximum allowed time or cut-off time.

$$\text{Percent protection }\left(\%\right)=\left(T_{t}-T_{0}\right)/\left(T_{m}-T_{0}\right)\times 100$$

#### 2.6.2 *In vivo* acetic acid-induced writhing test

Healthy swiss albino mice (n:30) of both sexes, weighing 20–30 g, were allocated into six groups at random of control, standard, and test as described in Section 2.5.2. Respective doses were administered to animals in each group and after 30 min, 10 mL/kg b of acetic acid (0.6%) was given intraperitoneally. Counting of abdominal writhes (constrictions) began 5 minutes afterward and continued for 20 min following the administration of acetic acid. The significant reduction in the average number of abdominal writhes in the treated groups against the control group was interpreted as evidence of the effectiveness of the anti-nociceptive agent. Percent protection of each group was calculated (Koster, 1959; Gupta et al., 2015) where Mean N_c_ refers to the mean number of abdominal writhes in the control group and Mean N_t_ refers to the mean number of abdominal writhes in the test group.

$$\text{Percent protection }\left(\%\right)=\left(\text{Mean }N_{c}\;–\text{ Mean }N_{t}\right)/\left(\text{Mean }N_{c}\;\right)\times 100$$

### 2.7 Antioxidant activity

#### 2.7.1 *In vitro* DPPH assay

Ethanolic DPPH of concentration 80 μg/mL (4mg/50 mL) was used to identify the antioxidant potential at 1, 1.5, 2.5, 5, 7.5, and 10 mM of MA-AgNPs (C). Control (DPPH in ethanol, 3:1) and standard ascorbic acid (3.80 mM) analyses were carried out simultaneously for comparison by adding 1 mL of DPPH to each test tube and ethanol as diluent to make up the final volume of 3 mL. Solutions were then allowed to rest in drak for 30 min during which the solution’s color changed from violet to yellow in the presence of antioxidants. The absorbance was determined at 517 nm (Blois, 1958) and radical scavenging activity was calculated in percentage by applying the following formula, where A_0_ is the absorbance of blank and A_1_ is the absorbance of sample.

$$\text{DPPH radical scavenging activity }\left(\%\right)=\left[\left(A_{0}\;–\;A_{1}\right)/\;A_{0}\right]\times 100$$

#### 2.7.2 *In vivo* antioxidant activity (SOD level determination)

SOD levels in the paw tissue of rats were examined to ascertain *in vivo* antioxidant effectiveness. Determination was made by performing RT-PCR as mentioned in section 2.5.3.

### 2.8 Statistical analysis

Statistical analysis was performed in SPSS 29. Data was presented as mean ± SD. One-way ANOVA followed by Dunnet’s test was applied for group comparison, keeping the significance level <0.05, and repeated measured ANOVA followed by LSD was applied in tests where readings were recorded at multiple time intervals. Regression analysis was performed in DPPH assay and Brine Shrimp Lethality Bioassay.

## 3 Results

### 3.1 Toxicity testing

#### 3.1.1 Acute toxicity testing

**FIGURE 1:**
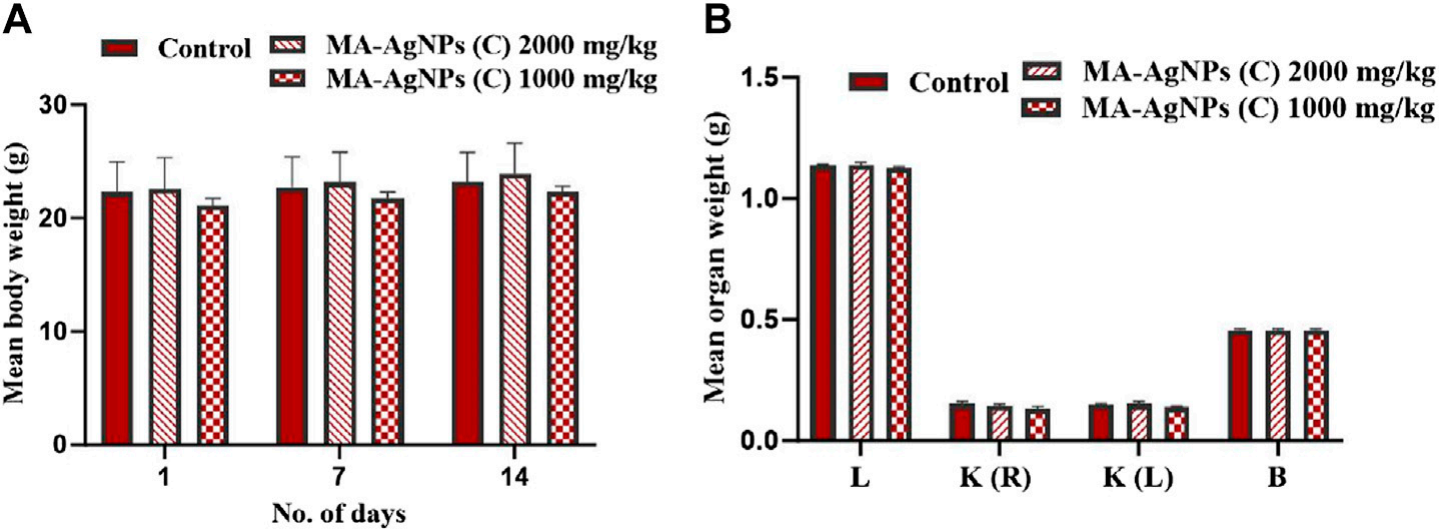
**(A)** Mean ± SD body weight of animals in control and test groups administered with 1000 and 2000 mg/kg MA-AgNPs (C) at 1, 7, and 14 days of acute toxicity testing. The difference in mean weight with control was statistically insignificant (*p*-value > 0.05). **(B)** Mean ± SD organ (liver, kidney, and brain) of control and treated (2000 and 1000 mg/kg) groups. Mean variations were not statistically significant (*p*-value > 0.05).

*In vivo,* acute toxicity of MA-AgNP s(C) was analyzed on swiss albino female mice at the dose of 2,000 mg/kg following OECD 423 guidelines. 14 days of regular monitoring revealed that there were no prominent toxic signs, and all the general clinical characteristics, behaviors, and symptoms were normal. Significant hazardous effects and fatalities were not found at the *p*-value > 0.05. All the animals under study remained alive till the last day of observation suggesting that the LD_50_ of MA-AgNPs (C) is greater than 2,000 mg/kg. The studied animal’s body weight (Figures 1A,B) and organ weights were recorded.

#### 3.1.2 Sub-acute toxicity testing

**FIGURE 2:**
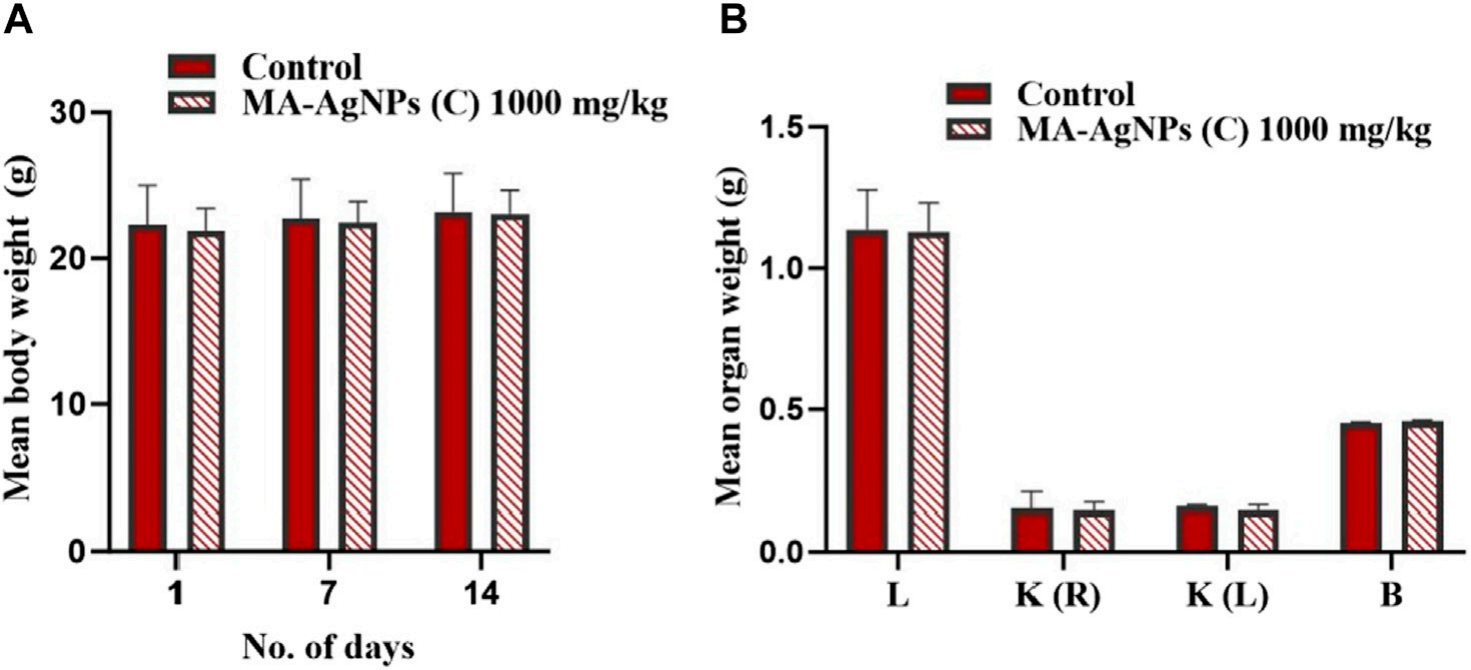
**(A)** Mean ± SD body weight of animals in control and test groups administered daily with 1000 mg/kg MA-AgNPs (C) at 1, 7, and 14 days of sub-acute acute toxicity testing. The difference in mean weight with control was statistically insignificant (*p*-value > 0.05). **(B)** Mean ± SD organ (liver, kidney, and brain) of control and treated (1000 mg/kg) groups. Mean variations with control were not statistically significant (*p*-value > 0.05).

Repeated dose oral toxicity testing was performed at the dose of 1,000 mg/kg. No significant toxic signs or fatalities were recorded during the 14-day observation period. Mean differences in animal and organ weights were determined (Figures 2A,B) and found to be statistically insignificant (*p*-value > 0.05).

#### 3.1.3 Histopathology

##### 3.1.3.1 Kidney

**FIGURE 3:**
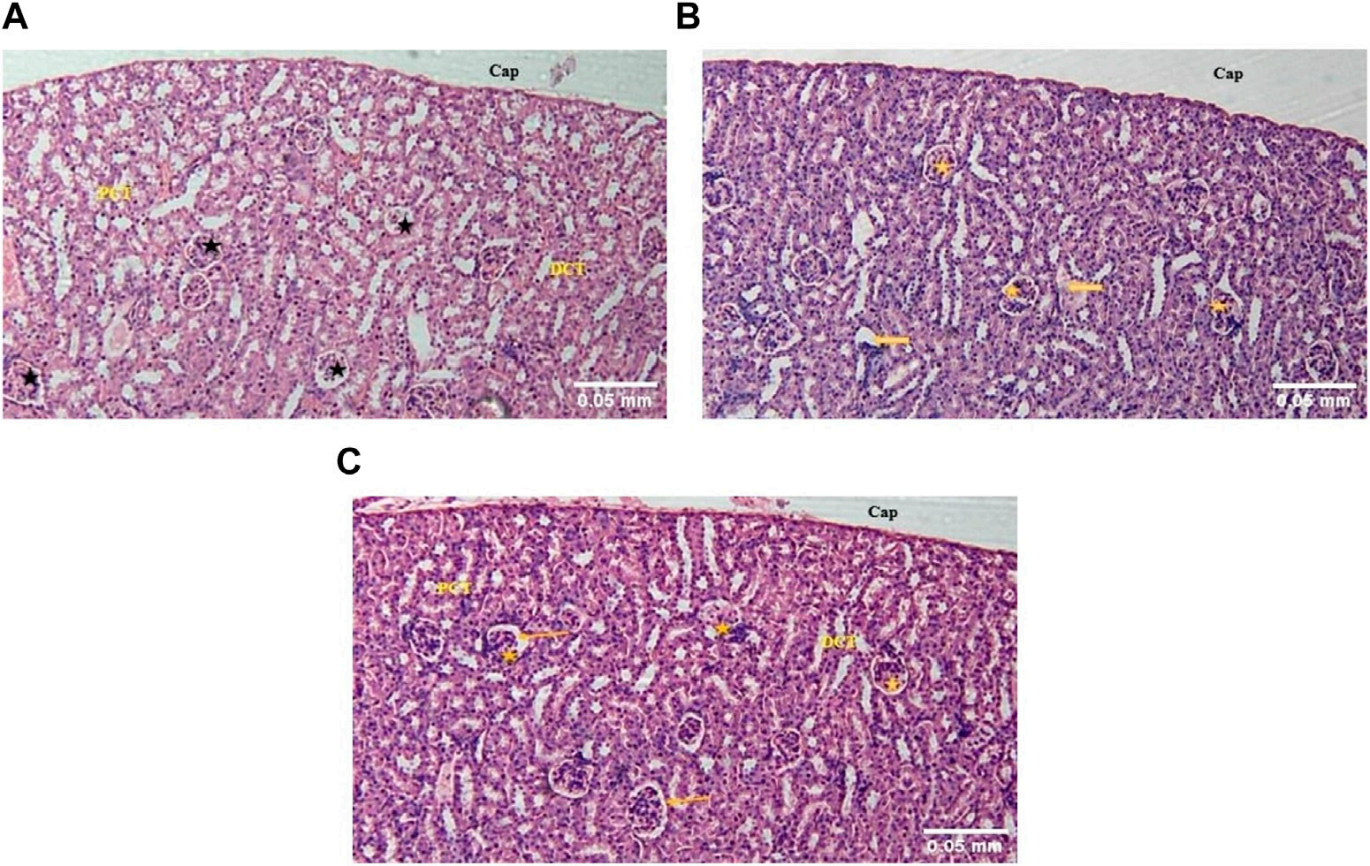
**(A)** The kidney of control mice showed a normal cortex lined by a thick fibrous connective tissue capsule (Cap) and numerous renal corpuscles (star). A flattened epithelium lining the Bowman’s capsule can be seen. Proximal convoluted tubules (PCT) with a brush border and distal convoluted tubules (DCT) are lined by a single layer of cuboidal cells. (H&E; 100X). **(B)** The kidney of acute toxicity mice treated with 2000 mg/kg MA-AgNPs **(C)** showed an almost normal cortex lined by a thick capsule (Cap) and numerous renal corpuscles (star). Few tubules seem to have desquamated epithelium and deranged structures (arrows). (H&E; 100X). **(C)** The kidney of Sub-acute toxicity mice treated with 1000 mg/kg MA-AgNPs **(C)** showed a cortex lined by thick fibrous connective tissue capsule (Cap) and abundant renal corpuscles (star). A flattened epithelium lining the Bowman’s capsule can be seen. A slightly widened urinary space can be observed (arrows). Proximal and distal convoluted tubules (PCT and DCT) are visible also. (H&E; 100X).

In the kidney of control mice, the normal architecture of the cortex and medulla was observed. The cortex, surrounded by a thick capsule, showed numerous normal renal corpuscles, with Bowman’s capsule and glomerular tuft of capillaries. The proximal and distal renal tubules were lined by simple cuboidal epithelium (Figure 3A). In the acute toxicity (MA-AgNPs (C) 2,000 mg/kg) treated group, few tubules appeared deranged with desquamation of their lining epithelium (Figure 3B). Apparently normal renal cortical and medullary architecture with a mild widening of the urinary space was noticed in sub-acute toxicity group (1,000 mg/kg MA-AgNPs (C)) (Figure 3C).

##### 3.1.3.2 Liver

**FIGURE 4:**
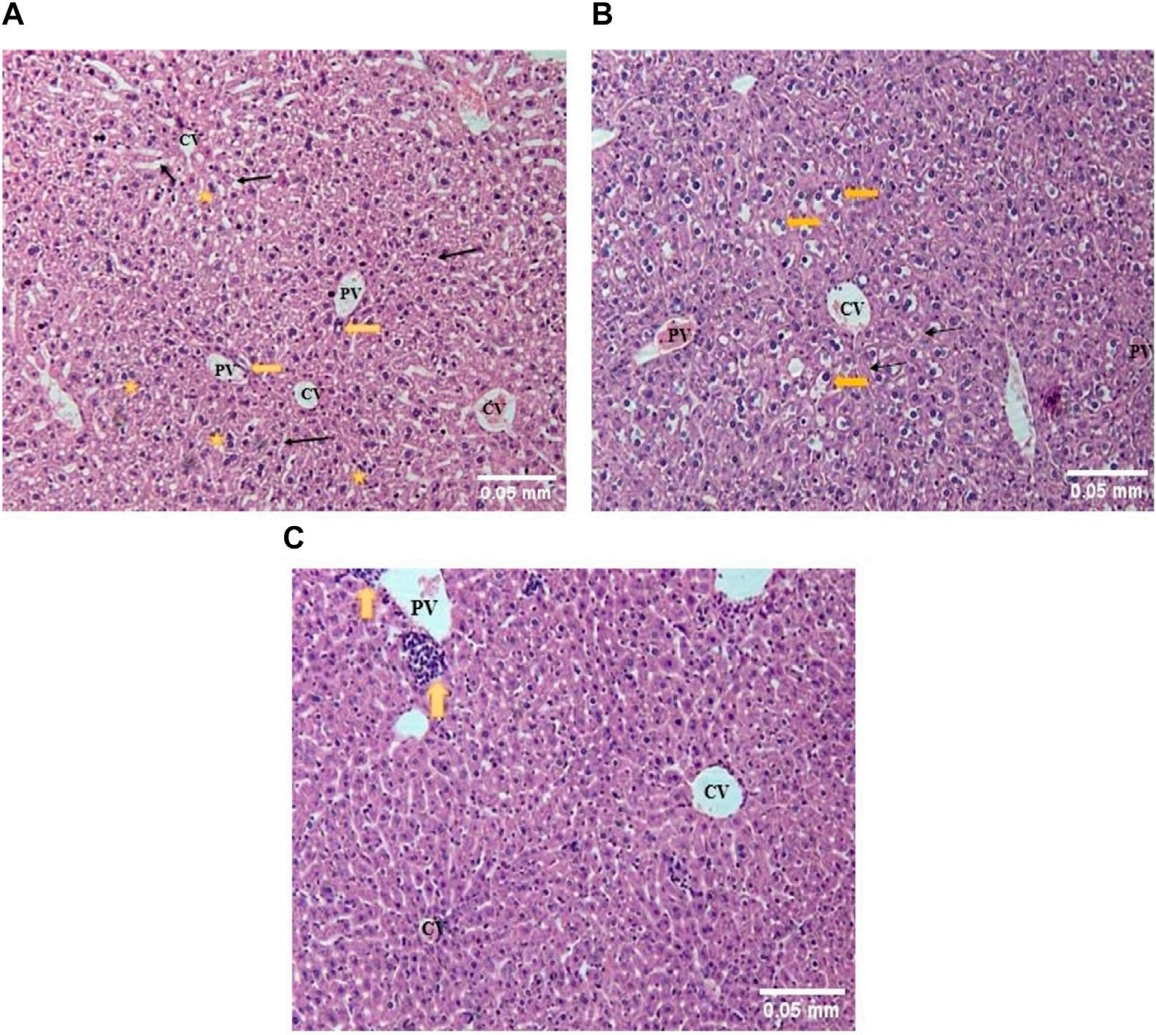
**(A)** The liver of control mice showed rows of hepatocytes radiating from the central vein (CV), with sinusoids (thin arrows) in between. The portal triad showed a portal vein (PV) and bile duct (thick arrow). Binucleated hepatocytes (stars) could also be seen. (H&E; 100X). **(B)** The liver of acute toxicity mice showed slightly deranged hepatic architecture with moderately swollen hepatocytes (yellow arrows) radiating from the central vein (CV), separated by narrowed sinusoids (thin arrows). The portal vein (PV) appeared congested. (H&E; 100X). **(C)** The liver of sub-acute toxicity mice showed almost normal hepatic architecture with a central vein (CV) surrounded by radial rows of apparently normal hepatocytes. Mononuclear leukocyte infiltration (yellow arrow) was observed near the portal vein (PV) (H&E; 100X).

In the liver of control mice, normal hepatic lobular architecture with a central vein and radiating rows of hepatocytes with sinusoids in between was observed. A few bi-nucleate hepatocytes were also seen. The portal triad contained a portal vein, bile duct lined by cuboidal epithelium, and hepatic artery (Figure 4A). In an acute toxicity group, the hydropic swelling of the hepatocytes increased, the sinusoidal spaces decreased, and the portal vein was dilated and congested (Figure 4B). In the sub-acute toxicity group, normal hepatic lobules were observed with mononuclear cell infiltration near the portal vein (Figure 4C).

##### 3.1.3.3 Brain

**FIGURE 5:**
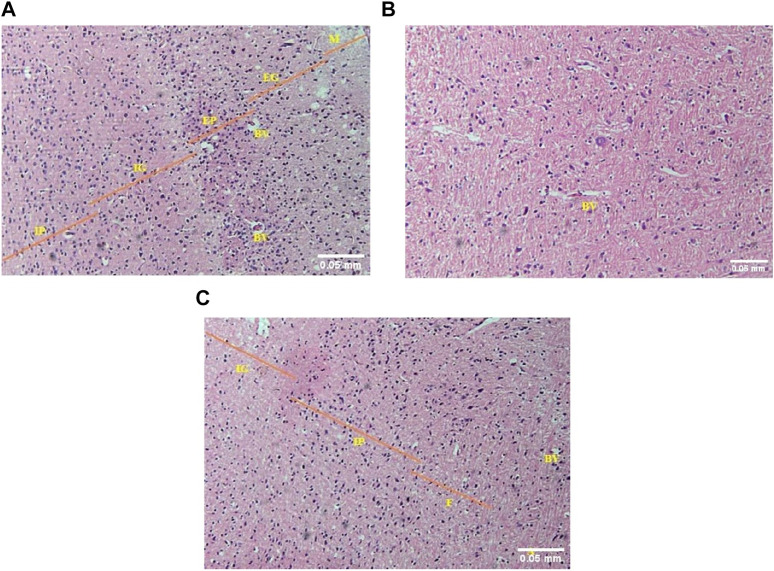
**(A)** The photomicrograph of the brain (cerebrum) of control mice showed M = molecular (plexiform), EG = external granular, EP = external pyramidal, IG = internal granular, and IP = internal pyramidal layers. BV = blood vessels (H&E; 100X). **(B)** The photomicrograph of the brain (cerebrum) of acute toxicity mice showed indiscernible cerebral layers but the neuronal cellularity was apparently near normal. Blood vessels = BV (H&E; 100X). **(C)** The photomicrograph of the sub-acute toxicity brain (cerebrum) showed IG = internal granular, IP = internal pyramidal, and F = multiform (fusiform) layers. The cerebral architecture appeared normal apparently. BV = blood vessels, A = white matter containing axons of neurons (H&E; 100X).

In the brain (cerebrum) of control mice, the normal histological arrangement of six different layers was observed as follows: molecular (plexiform), external granular, external pyramidal, internal granular, internal pyramidal and multiform (fusiform) layer (from outside to inside) (Figure 5A). In the acute toxicity group, although the neuronal density seemed normal, the cerebral layers were not indiscernible (Figure 5B). Apparently normal cerebral architecture was seen in the sub-acute toxicity group and normal cerebral layers were observed (Figure 5C).

#### 3.1.4 Brine Shrimp Lethality Bioassay

The number of floating nauplii that remained alive after 24 h was counted and 20% mortality was recorded at the highest concentration of MA-AgNPs (C) (Table 2) i.e., at 169.8 μg/mL, whereas at 85 μg/mL 0% mortality was seen. Vincristine sulphate was found to have LC_50_ of 0.753 μg/mL, while MA-AgNPs (C) had LC_50_ of 38062.7 μg/ml based on interpolation of the regression plot.

**TABLE 2:** Probit of kill and percentage mortality of AgNPs (C) and VS at different concentrations alone.

Test sample	Volume (µg/ml)		log concentration	Percent mortality	Probit of kill	LC_50_ (µg/mL)
**MA-AgNPs (C)**	0.84	0.6989		0	-	38062.7
	4.24	1.3979		0	-	
	8.50	1.6989		0	-	
	17.0	2		0	-	
	84.9	2.6989		0	-	
	169.8	3		20	4.16	
**Vincristine sulfate**	0.06	−1.2218		10	3.72	0.753
	0.125	−0.9031		20	4.16	
	0.25	−0.6021		20	4.16	
	0.50	−0.301		40	4.75	
	1.00	0		50	5	
	5.00	0.698		90	6.28	
	10.0	1		100	7.33	

### 3.2 Anti-inflammatory activity

#### 3.2.1 *In vitro* protein denaturation method

*In vitro,* the anti-inflammatory efficiency of MA-AgNPs (C) was identified alone and in synergism with standard through the protein denaturation method. The mean ± SD absorbance of each group recorded through the spectrophotometer is presented in Table 3. Mean comparisons of AgNPs (C) alone at 425 and 850 μg/mL were found statistically highly significant (*p*-value<0.001). Percentage protein denaturation inhibition calculated through formula revealed that MA-AgNPs (C) inhibited denaturation by 46.14% and 61.48% at 425 and 850 μg/mL. Diclofenac Na alone had a percentage inhibition of 48.26 (*p*-value < 0.001). Synergism of MA-AgNPs (C) with standard showed highly significant inhibition with the highest percentage inhibition of 63.71% and 72.09% respectively.

**TABLE 3:** *In vitro* protein denaturation of the MA-AgNPs (C) alone and in synergism with studied anti-inflammatory agents at different concentrations.

Groups	Mean ± SD absorbance	Percent inhibition (%)	*p*-value
**Distilled water (control)**	0.725 ± 0.026	-	-
**MA-AgNPs (C) 425** ** ** **μg/mL**	0.390 ± 0.049	46.14^***^	0.000
**MA-AgNPs (C) 850** ** ** **μg/mL**	0.279 ± 0.037	61.48^***^	0.000
**Diclofenac Na (1000** ** ** **μg/mL)**	0.375 ± 0.061	48.26^***^	0.000
**MA-AgNPs (C) 425** ** ** **μg/mL +1000** ** ** **μg/mL Diclofenac Na**	0.263 ± 0.106	63.71^***^	0.000
**MA-AgNPs (C) 850** ** ** **μg/mL +1000** ** ** **μg/mL Diclofenac Na**	0.202 ± 0.026	72.09^***^	0.000

*p*-value significant at ≤0.05. *p-value ≤ 0.05, ** *p*-value < 0.01, ***p-value < 0.001.

#### 3.2.2 *In vivo* anti-inflammatory activity (carrageenan-induced paw edema method)

**FIGURE 6:**
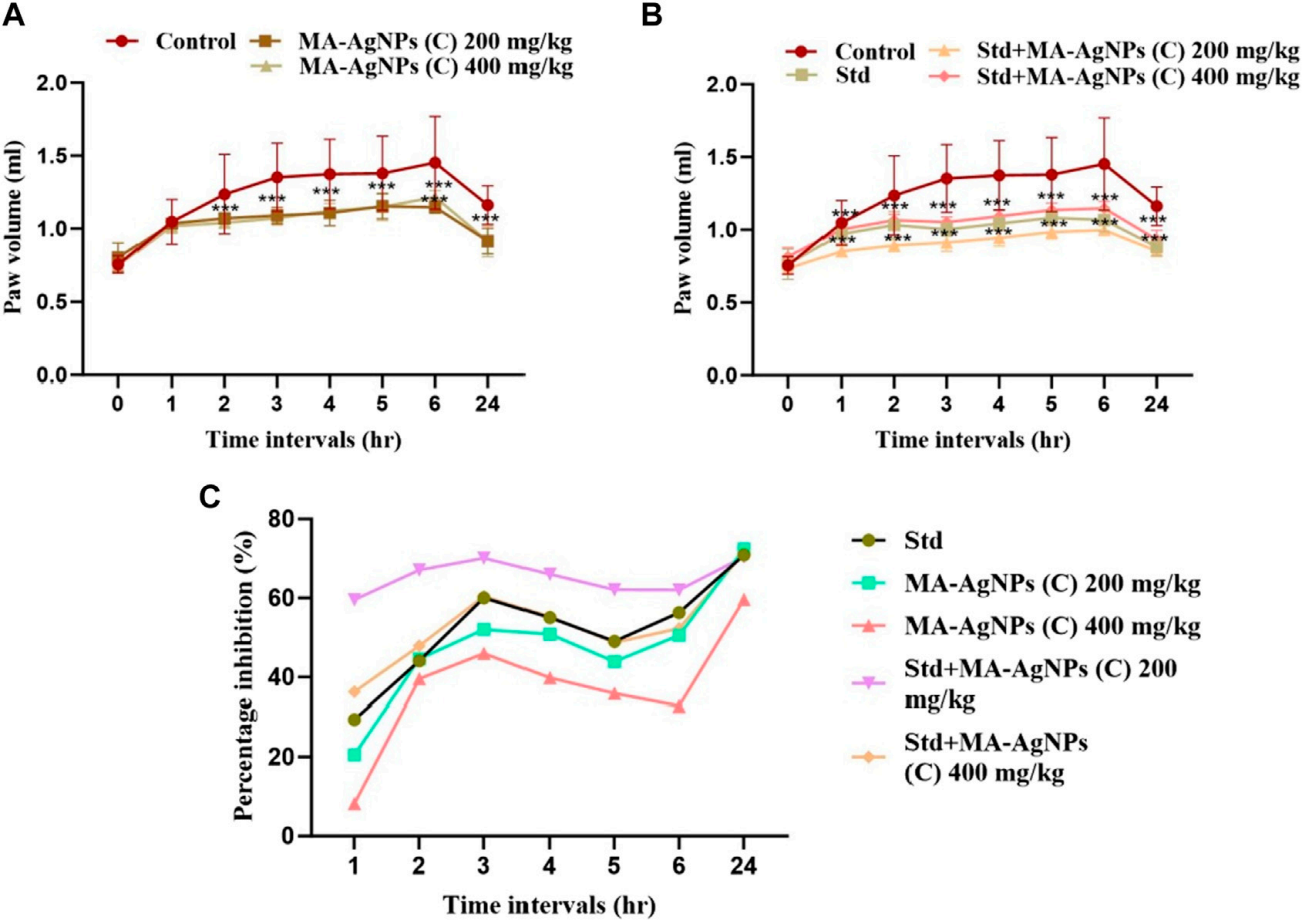
*In vivo* anti-inflammatory efficiency of MA-AgNPs (C) was determined by the carrageenan paw edema method. **(A)** represents mean ± SD paw volume of control and MA-AgNPs (C) (200 and 400 mg/kg) at 1, 2, 3, 4, 5, 6, and 24 h post carrageenan administration. **(B)** represents the mean ± SD of control, standard, and synergistic groups (200 and 400 mg/kg). **(C)** represents the percentage inhibition of edema of groups at different time intervals. *p-value < 0.0014, ** *p*-value < 0.0010, ***p-value < 0.0005.

*In vivo,* the anti-inflammatory activity of MA-AgNPs (C) alone as well as in synergism with the standard was analyzed through the carrageenan-induced paw edema method by determining the paw volume -. Significant paw edema reduction (*p* < 0.001) was noticed in rats administered with MA-AgNPs (C) (200 and 400 mg/kg) at 2, 3, 4, 5, 6, and 24 h post carrageenan administration with highest percentage inhibition of 72.41% and 59.6% noticed at 24th hour (Figure 6). Similarly prominent anti-inflammatory efficiency (*p*-value < 0.001) has been identified in synergistic groups of 200 and 400 mg/kg with standard at 2, 3, 4, 5, 6, and 24 h post carrageenan with the highest percentage inhibition of 70.44% and 71.42% respectively observed at 24th hour while that of standard alone was 70.93%.

#### 3.2.3 Molecular marker identification by measurement of tissue inflammatory cytokines

##### 3.2.3.1 Gene expression of TNF-α

Enhanced gene expression of TNF-α was recorded in the control group whereas reduced levels were observed in the treated groups at both doses of MA-AgNPs (C) which was statistically significant (*p*-value<0.05). However, reduction was more pronounced in synergistic groups (Table 4).

**TABLE 4:** Gene expression of TNF-α at the studied doses of MA-AgNPs (C) alone and in synergism with standard studied drugs.

MA-AgNPs (C) (n = 5)	Mean ± SD	*p*-value
**Control**	323.37 ± 1.44	
**Standard**	40.755 ± 0.77^**^	0.0014
**200** ** ** **mg/kg**	43.745 ± 0.77^**^	0.0042
**400** ** ** **mg/kg**	111.79 ± 1.13^**^	0.0093
**Std + 200** ** ** **mg/kg**	32.325 ± 0.67^**^	0.0008
**Std + 400** ** ** **mg/kg**	1.7450 ± 0.20^***^	0.0000

*p*-value significant at ≤0.05. *p-value ≤ 0.05, ** *p*-value < 0.01, ***p-value < 0.001.

##### 3.2.3.2 Gene expression of IL-6

Significantly lower gene expression of IL-6 was recorded in the groups treated with either lower or higher doses of MA-AgNPs (C) (*p*-value < 0.05) similar to that of TNF-α. In both instances, the reduction was again noticeable in synergistic groups (Table 5).

**TABLE 5:** Gene expression of IL-6 at the studied doses of MA-AgNPs (C) alone and in synergism with standard studied drugs.

Sample (n = 5)	Mean ± SD	*p*-value
**Control**	326.37 ± 0.78	
**Standard**	40.750 ± 0.77^**^	0.0024
**200** ** ** **mg/kg MA-AgNPs (C)**	43.741 ± 0.85^**^	0.0042
**400** ** ** **mg/kg MA-AgNPs (C)**	142.79 ± 2.55^**^	0.0073
**Std + 200** ** ** **mg/kg MA-AgNPs (C)**	32.321 ± 0.67^**^	0.0007
**Std + 400** ** ** **mg/kg MA-AgNPs (C)**	1.7401 ± 0.205^***^	0.0000

*p*-value significant at ≤0.05. *p-value ≤ 0.05, ** *p*-value < 0.01, ***p-value < 0.001.

### 3.3 Antinociceptive activity

#### 3.3.1 Tail flick test

**FIGURE 7:**
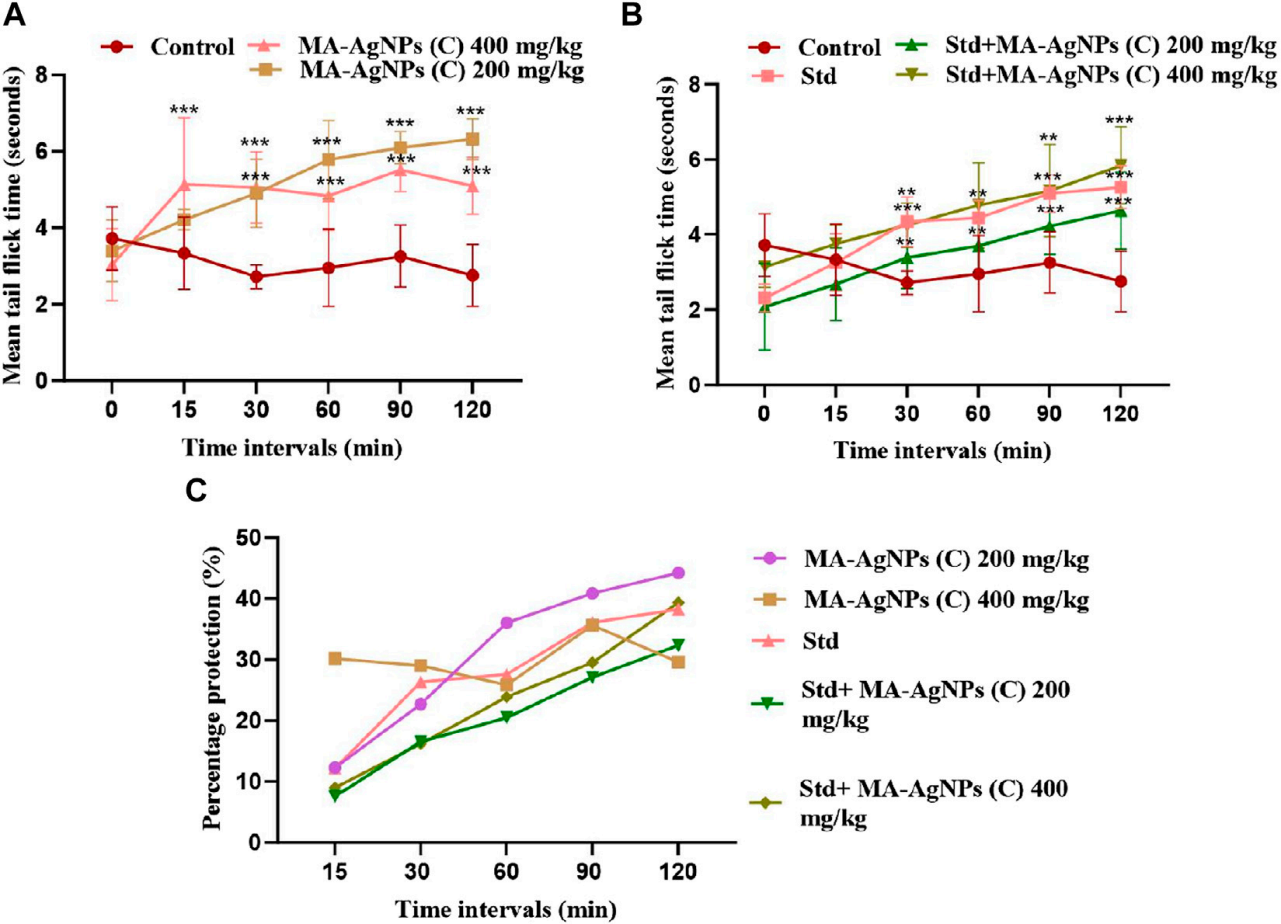
Represents *in vivo* anti-nociceptive efficiency of MA-AgNPs (C) determined by the tail flick method. **(A)** represents mean ± SD tail flick time of control and MA-AgNPs (C) (200 and 400 mg/kg) at 15, 30, 60-, 90-, and 120 min post-drug administration. **(B)** represents the mean ± SD of control, standard, and synergistic groups (200 and 400 mg/kg). **(C)** represents the percentage protection of pain at different time intervals. *p-value < 0.002, ** *p*-value < 0.0015, ***p-value < 0.0010.

Tail flick response of animals in each group was recorded before drug administration (T_0_) as well as at 15, 30, 60, 90, and 120 min post-drug administration. Maximum pain inhibition of MA-AgNPs (C) (200 and 400 mg/kg alone) is noticed at 120 min as 44.24% and 29.59% respectively. While in synergism with standard 32.34% and 39.35% were the highest responses recorded at 120 min. Standard alone showed percentage protection of 38.28% at 120 min. Statistically significant results of all groups have been obtained at 90, and 120 min having *p*-value < 0.01 (Figure 7).

#### 3.3.2 Acetic acid induced writhing test

MA-AgNPs (C) showed a statistically significant reduction in writhe count at the doses of 200 mg/kg and 400 mg/kg (*p*-value = 0.000, i.e., <0.001) with percentage protection of 57.32% and 63.41% respectively. Standard (Diclofenac Na 50 mg/kg) alone also showed a significant reduction with a percentage protection of 69.51%. Synergism of 200 mg/kg and 400 mg/kg MA-AgNPs (C) along with standard showed percentage protection of 85.36% and 76.82% both of which were statistically highly significant (*p*-values < 0.001) (Table 6).

**TABLE 6:** Acetic acid induced writhe count of groups at the studied doses of MA-AgNPs (C) alone and in synergism with standard studied drugs.

Groups (n = 5)	Mean ± SD No of writhes	Percentage protection (%)	Mean difference (*p*-value)
**Control**	16.4 ± 1.14	-	-
**Standard**	5 ± 1	69.51^***^	11.40 (0.000)
**200** ** ** **mg/kg MA-AgNPs (C)**	7 ± 1	57.32^***^	09.40 (0.000)
**400** ** ** **mg/kg MA-AgNPs (C)**	6 ± 1	63.41^***^	10.40 (0.000)
**Standard + 200** ** ** **mg/kg MA-AgNPs (C)**	2.4 ± 0.54	85.36^***^	14.00 (0.000)
**Standard + 400** ** ** **mg/kg MA-AgNPs (C)**	3.8 ± 0.83	76.82^***^	12.60 (0.000)

*p*-value significant at ≤0.05. *p-value ≤ 0.05, ** *p*-value < 0.01, ***p-value < 0.001.

### 3.4 Antioxidant activity

#### 3.4.1 *In vitro* antioxidant activity (DPPH assay)

**FIGURE 8:**
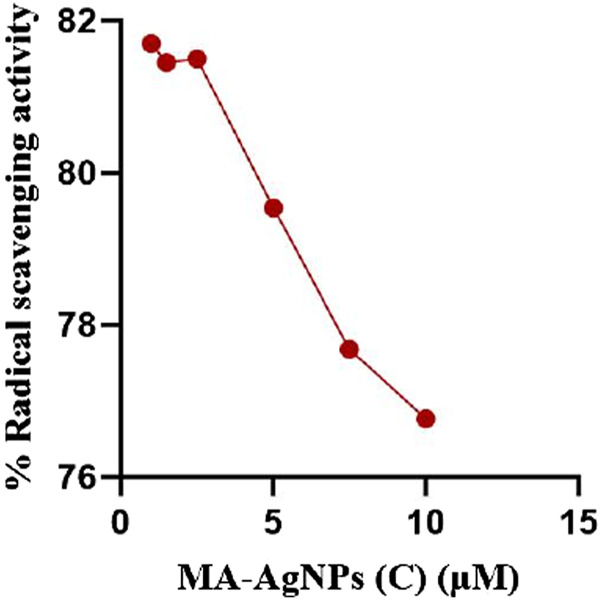
*In vitro* antioxidant potential of MA-AgNPs (C) examined through DPPH assay. % age radical scavenging activity is higher at lower concentrations and decreases with increasing concentration.

*In vitro* antioxidant activity of MA-AgNPs (C) has been analyzed through DPPH. Significant antioxidant potential has been identified and percentage radical scavenging activities was found to be decreasing with increasing concentration. The highest activity was recorded at the lowest concentration, i.e. 1 mM 81.70% while the least response was noticed at the highest concentration, i.e. 10 mM (Figure 8) and the calculated IC_50_ value was 55.094 mM.

#### 3.4.2 *In vivo* estimation of superoxide dismutase (SOD) levels

Lower expression of SOD was recorded in treated groups than control groups (Table 7) but the comparison was statistically insignificant (*p*-value > 0.05).

**TABLE 7:** Gene expression of MA-AgNPs (C) alone and in synergism with standard studied drug against SOD.

Sample (n = 5)	Mean ± SD	*p*-value
**Control**	82.87 ± 2.07	
**Standard**	55.23 ± 2.17^*^	0.023
**200** ** ** **mg/kg MA-AgNPs (C)**	45.50 ± 1.803	0.152
**400** ** ** **mg/kg MA-AgNPs (C)**	66.67 ± 2.05	0.312
**Std + 200** ** ** **mg/kg MA-AgNPs (C)**	31.06 ± 1.89^**^	0.008
**Std + 400** ** ** **mg/kg MA-AgNPs (C)**	25.59 ± 2.06	0.156

*p*-value significant at ≤0.05. *p-value ≤ 0.05, ** *p*-value < 0.01, ***p-value < 0.001.

## 4 Discussion

Inflammation is a defensive host response but unfortunately, deregulation and augmentation of this protective host response may lead to several medical ailments (Murakami et al., 2013; Gupta et al., 2015; Hirano, 2021). Clinicians generally recommend steroidal and non-steroidal anti-inflammatory medicines to treat mild, moderate, or chronic inflammatory illnesses. However, these medications have a variety of dose-dependent adverse drug reactions (Nesbitt, 1995; Kim et al., 2009). Chronic inflammatory disorders are considered a leading cause of mortality globally; therefore it is imperative to discover novel therapeutic agents.

Silver metal has been valued as a potent medicinal substance since ancient times due to its extensive clinical relevance. Moreover, the emergence of nanotechnology has brought attention to its significance (Alexander, 2009; Barillo and Marx, 2014; Medici et al., 2019). Reagents utilized for the synthesis of nanoparticles and experimental conditions strongly influence the physical, chemical, and pharmacological activities of AgNPs (Wei et al., 2015; Castro-Aceituno et al., 2016). The current research focused on identifying the anti-inflammatory, antinociceptive, and antioxidant efficiencies of MA-AgNPs (C) utilizing *in vitro* and *in vivo* approaches. Toxicity profiling of these AgNPs has not been identified yet hence before approaching towards exploring their therapeutic potential, toxicity was analyzed through acute and sub-acute toxicity testing while cytotoxicity evaluation was done by performing brine shrimp lethality.

### 4.1 Toxicity test

Considering the published studies, the LD_50_ of MA-AgNPs synthesized using diverse techniques and utilizing different reagents is greater than the dose of 2,000 mg/kg body weight (Maneewattanapinyo et al., 2011; Jacob et al., 2017; Padilla-Camberos et al., 2022). Hence, acute toxicity of MA-AgNPs (C) was identified by performing a limit test at 2,000 mg/kg. Prominent adverse reactions and mortalities were not seen; additionally, the weight variations with control were statistically insignificant. Results supported the established literature that the LD_50_ of MA-AgNPs (C) is greater than 2,000 mg/kg (Maneewattanapinyo et al., 2011; Jacob et al., 2017; Padilla-Camberos et al., 2022). Similarly, findings of repeated dose oral toxicity testing performed at 1,000 mg/kg (Ansari et al., 2016) revealed similar results, and significant toxic signs and mortalities were not recorded. These findings pointed towards the safety of MA-AgNPs which agrees with earlier published research (Maneewattanapinyo et al., 2011; Alwan et al., 2021). Nevertheless, some studies claim the toxicity AgNPs in terms of accumulation in vital organs (Xue et al., 2012; Recordati et al., 2021). These contradictory *in vivo* toxicity findings are due to the AgNP’s variance in terms of size, dose, coating and dispersion state, administration technique, exposure period, production method, and reagents utilized (Skebo et al., 2007).

### 4.2 Brine shrimp lethality bioassay.

Brine shrimp lethality cytotoxicity analysis was determined at 0.84, 4.24, 8.5, 17, 84.9, and 169.8 μg/mL which were selected after conducting a comprehensive literature review (Dilshad et al., 2020). A lethality of 20% was identified at the highest concentration of 169.8 μg/mL with LC_50_ value of 38062.7 μg/ml and cytotoxicity concentration corresponding to the published articles (Kumar et al., 2012; Arulvasu et al., 2014). The findings of Yi Low S et al. and Singh R. showed that LC_50_ of AgNPs is recorded at higher concentrations (Low et al., 2022; Singh et al., 2022). Whereas other research revealed that the cytotoxicity (LC_50_) of AgNPs made using different approaches and reagents was seen at lower concentrations, i.e., 4 nM (Arulvasu et al., 2014), 50 μg/mL (Priyaragini et al., 2013), 518 μg/mL (Asghar et al., 2020). The discrepancy across studies is once again referring to the assumption that the therapeutic potential of AgNPs differs depending on the source, synthesis method, and the manufacturing reagents used for them (Van der Zande et al., 2012).

### 4.3 *In vitro* anti-inflammatory efficacy

The protein denaturation method was employed to assess the *in vitro* anti-inflammatory efficacy. Response was monitored at the concentrations of 425 and 850 μg/mL. Analysis revealed that the anti-inflammatory potential of MA-AgNPs (C) was nearly like diclofenac Na (1,000 μg/mL) at 425 μg/mL while a greater percentage of protein denaturation inhibition was noticed at 850 μg/mL. Furthermore, the effectiveness was potentiated when synergized with Diclofenac Na at both concentrations. The findings were consistent with past studies that the AgNPs possess prominent concentration-dependent *in vitro* anti-inflammatory response (Giridharan et al., 2014; Das et al., 2019; Prabakaran and Mani, 2019; Revathi and Dhanaraj, 2019; Vijayakumar et al., 2019; Anwar et al., 2021).

### 4.4 *In vivo* anti-inflammatory activity

MA-AgNPs (C) have been shown to possess marked *in vivo* anti-inflammatory activity that was identified by performing the carrageenan-induced paw edema method. Doses were selected based on an extensive literature review (Ansari et al., 2016; Dakhil, 2017; Gul et al., 2021). A dose of 200 and 400 mg/kg was decided to analyze *in vivo* anti-inflammatory and antinociceptive responses. Higher effectiveness has been recorded at lower doses, i.e., 200 mg/kg and it was nearly similar to standard Diclofenac Na 50 mg/kg while percentage paw edema inhibition was more pronounced when they were synergistically administered with standard. The carrageenan-induced paw edema model is regarded as an acute inflammation model that can identify the anti-inflammatory effects of medications that work by inhibiting cyclooxygenase. Consequently, it can be inferred that the likely mechanism underlying the MA-AgNPs (C)’ inhibitory impact on carrageenan-induced inflammation is suppression of the COX enzyme, which in turn inhibits the production of pro-inflammatory prostaglandin (Martelli, 1973). These results aligned with those of the earlier published articles. The *in vivo* anti-inflammatory potential of AgNPs synthesized by different physical and chemical approaches has already been reported (Ramachandran and Nair, 2011; David et al., 2014; El-Rafie and Hamed, 2014; Ahmad et al., 2015; Das et al., 2017; Kumaran, 2017; Ovais et al., 2018; Ammar et al., 2021). The synergistic activity observed in this study is endorsed by previously published articles in the same aspect. (Gupta et al., 2014; Alkawareek et al., 2019; Begum et al., 2022; Cao et al., 2022).

### 4.5 *In vivo* antinociceptive activity

*In vivo,* antinociceptive activity has been -inquired by both centrally and peripherally acting analgesic animal models, i.e., tail flick and acetic acid-induced pain models to identify their antinociceptive efficiency and possible mechanism. Both tests showed significant (*p*-value < 0.05) *in vivo* analgesic potential with higher percentage protection observed at lower doses complying with the results of *in vivo* carrageenan-induced paw edema method. Moreover, the synergistic response has been observed when these nanoparticles were concomitantly administered with standard drugs. Findings were in line with already reported studies (Khan et al., 2020; Rauf et al., 2021; Asad et al., 2022; Bozkaya et al., 2023) conducted by Ahmed N et al., reporting the anti-inflammatory and analgesic activity of AgNPs synthesized from Rosa damacena (Ahmad et al., 2015). Studies performed by Ilahi et al., 2021, Khattak U and co-workers (Khattak et al., 2019), Rauf et al., 2021, and some other researchers have identified the analgesic potential of AgNPs synthesized from different techniques, utilizing different reagents and experimental conditions (Bawazeer et al., 2022; Bozkaya et al., 2023).

### 4.6 *In vitro* and *in vivo* antioxidant activity

The imbalance between oxidants and antioxidants could result in increased levels of free radicals which may directly harm cellular elements through oxidation or an imbalance in the protease/antiprotease system. In addition to inducing cytotoxicity, elevated oxidative stress promotes the overexpression of genes that code for pro-inflammatory cytokines, which leads to increased inflammation. DPPH assay was performed to scrutinize the *in vitro* antioxidant potential at different concentrations of MA-AgNPs (C). The highest radical scavenging activity (81.7%) was observed at the lowest concentration (1 mM) while the least response was noticed at the highest concentration (10 mM) endorsing the aforementioned analgesic and anti-inflammatory response. -. Numerous studies have revealed that AgNPs have *in vitro* antioxidant activity, which supports our findings (Mittal et al., 2012; Priya et al., 2016; Selvan et al., 2018; Sreelekha et al., 2021; Begum et al., 2022). RT-PCR showed reduced SOD levels in groups treated with MA-AgNPs (C) in comparison to the control group. Greater SOD in the control group was indicative of enhanced inflammation that was suppressed in treated groups. Earlier studies have supported the same idea (Flynn and Melov, 2013; Leung et al., 2020).

Overall, this can be inferred that MA-AgNPs (C) can be used as efficient agents in reducing inflammation, pain, and oxidative stress. Responses were augmented when coadministered with standard drugs giving the idea of their synergistic potential. However, further investigations are required to explore the underlying mechanism behind their therapeutic responses.

## Data Availability

The datasets presented in this study can be found in online repositories. The names of the repository/repositories and accession number(s) can be found in the article/Supplementary material.
