# Supplementary material for: Molecular marker identification, antioxidant, antinociceptive, and anti-inflammatory responsiveness of malonic acid capped silver nanoparticle
Source: Front Pharmacol. 2024 Jan 31;14:1319613. doi: 10.3389/fphar.2023.1319613 (PMC10864560; doi:10.3389/fphar.2023.1319613)

**Supplementary files**

**S1:** Scheme 2. The synthesis mechanism of silver nanoparticles by using C-AgNPs.

**S2:** Brine Shrimp Lethality bioassay.

**S3:** Carrageenan-induced paw edema method.

**S4:** Tail Flick Method.

**S5:** list of abbreviations

**S6:** Ethical approval letters.


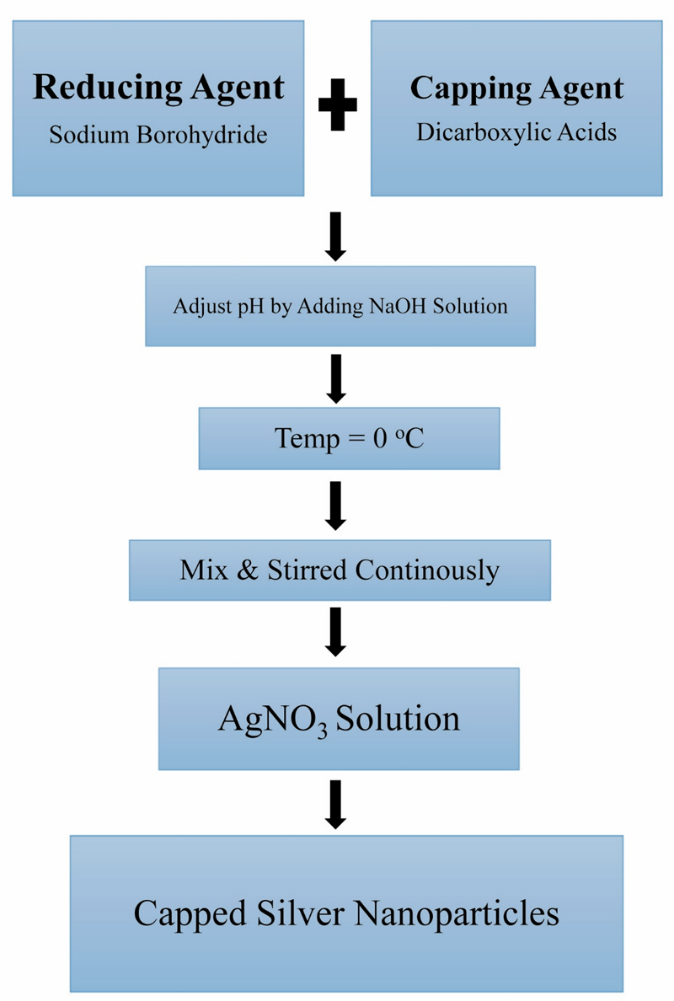


Scheme 2. The synthesis mechanism of silver nanoparticles by using C AgNPs


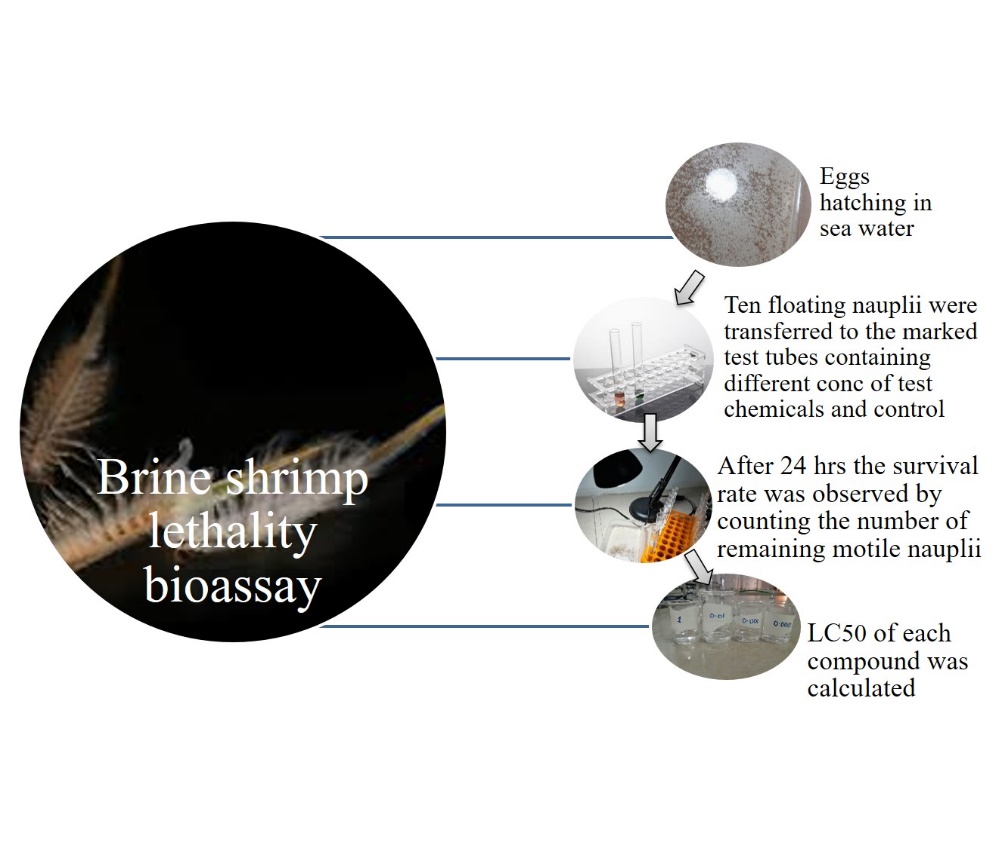


**S2: Brine Shrimp Lethality bioassay**


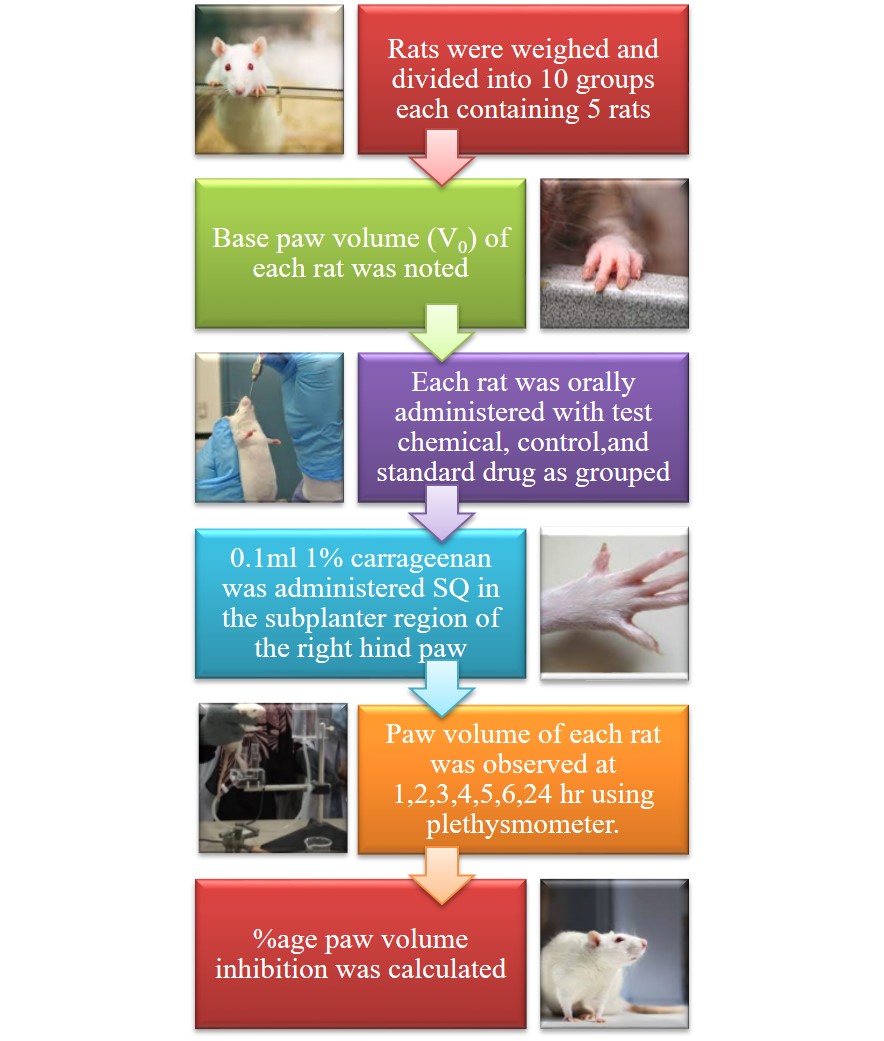


**S3: Carrageenan induced paw edema method**


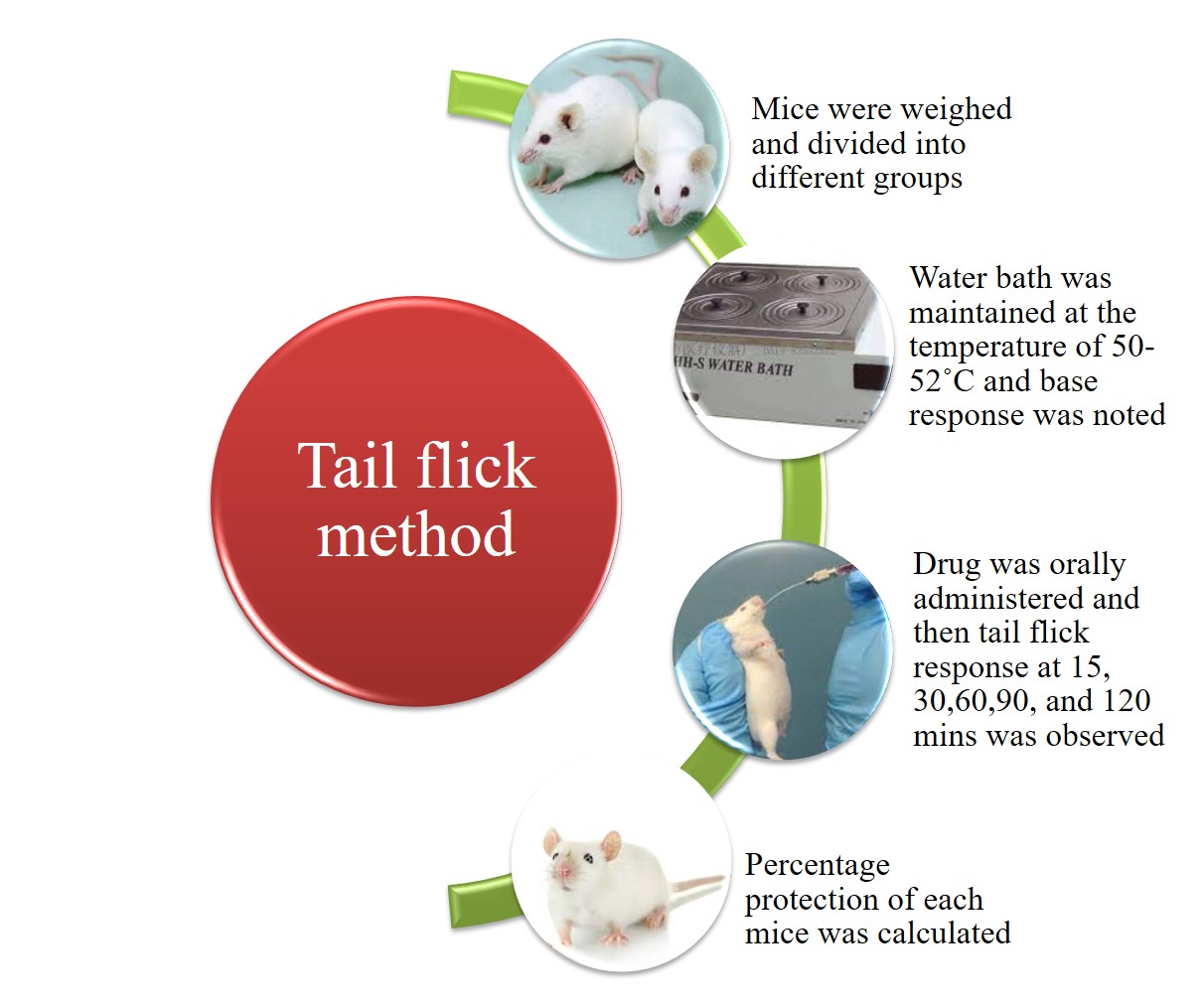


**S4: Tail Flick Method**

**S5: LIST OF ABBREVIATIONS**

˚C Degree Celcius

µg Microgram

µl Microliter

µl Microliter

µM Micromolar

Ag Silver

AgNO_3_ Silver Nitrate

AgNPs Silver Nanoparticles

AgNPs (C) Sodium Borohydride Malonic acid capped Silver Nanoparticles

ANOVA Analysis of variance

COX-2 Cyclooxygenase 2 enzyme

DLS Dynamic Light Scattering

DNA Deoxyribonucleic Acid

DPPH 2, 2 diphenyl-1-picrylhydrazyl

FT-IR Fourier Transform Infrared Spectroscopy

g Gram

GI Gastrointestinal

Gram +ve Gram-positive

Gram –ve Gram-negative

H&E Hematoxylin and Eosin

IC_50_ Half maximal inhibitory concentration

IL-6 Interleukin-6

IP Intraperitoneal

LC_50_ Lethal Concentration 50

LD_50_ Lethal Dose 50

M Molar

Max Maximum

Mg Milligram

MIC Minimum Effective Concentration

Min Minimum

Ml Milliliter

mM Millimolar

NaBH_4_ AgNPs Sodium Borohydride Silver Nanoparticles

NFκB Nuclear Factor Kappa B

Nm Nanometer

NO Nitric Oxide

NSAIDs Non-Steroidal Anti-Inflammatory Drugs

OECD Organization of Economic Co-operation and Development

PBS Phosphate Buffer Saline

PGE2 Prostaglandin E2

pH Potential of Hydrogen

RM ANOVA Repeated Measure Analysis of Variance

ROS Reactive Oxygen Species

RT-PCR Real-Time Polymerase Chain Reaction

SD Standard Deviation

SEM Scanning Electron Microscope

SOD Superoxide Dismutase

SPSS Statistical Package for Social Sciences

Std Standard

TAC Total Antioxidant Capacity

TCA Trichloroacetic Acid

TEM Transmission Electron Microscopy

TNF-α Tumor Necrosis Factor alpha

TSC AgNPs Trisodium citrate Silver Nanoparticles

UV visible Ultraviolet-visible

VS Vincristine Sulfate


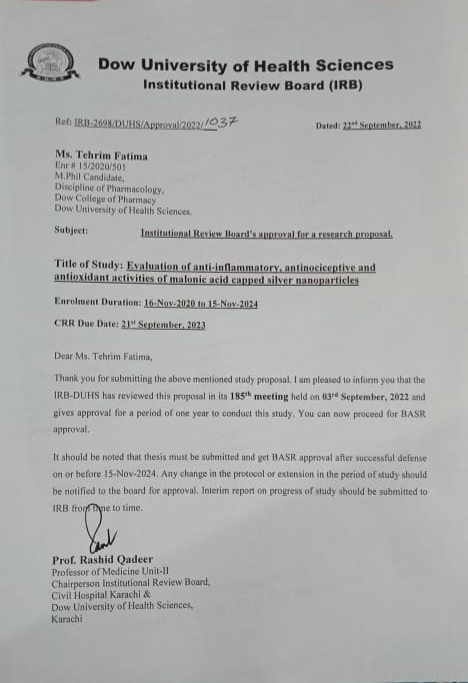

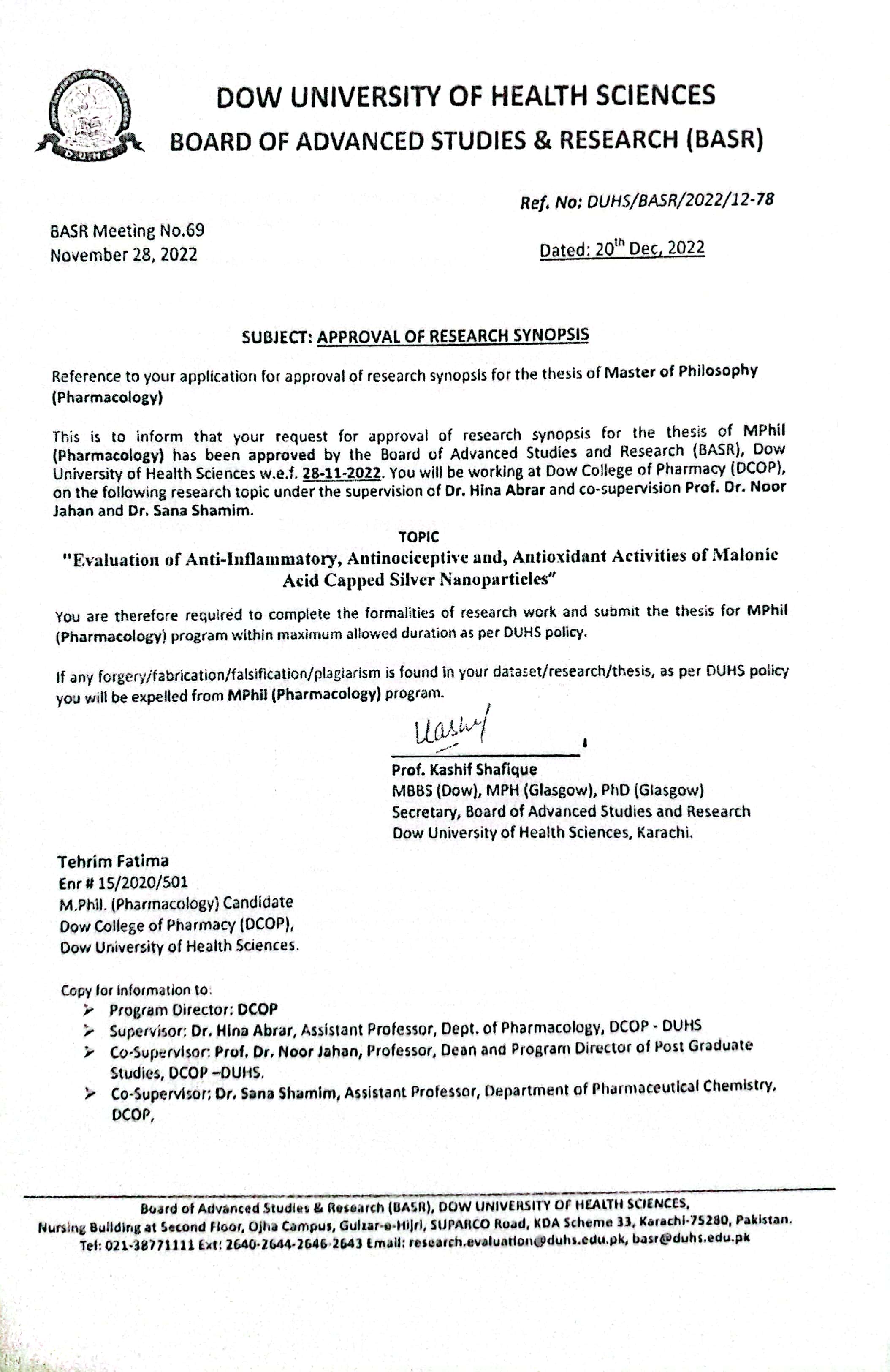

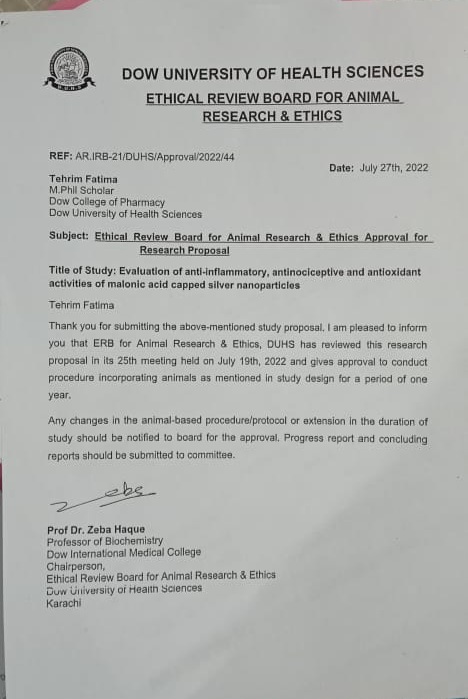

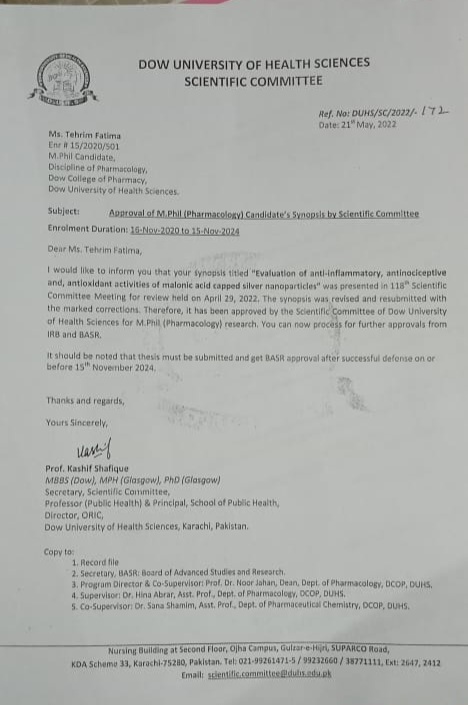

Supplement: Supplementary file 1 [file Table1.DOCX]
