# Supplementary material for: Molecular marker identification, antioxidant, antinociceptive, and anti-inflammatory responsiveness of malonic acid capped silver nanoparticle
Source: Front Pharmacol. 2024 Jan 31;14:1319613. doi: 10.3389/fphar.2023.1319613 (PMC10864560; doi:10.3389/fphar.2023.1319613)
Supplement: Supplementary file 2 [file DataSheet1.PDF]

Supplementary files

S1: Scheme 2. The synthesis mechanism of silver nanoparticles by using C-AgNPs.

S2: Brine Shrimp Lethality bioassay.

S3: Carrageenan-induced paw edema method.

S4: Tail Flick Method.

S5: list of abbreviations

S6: Ethical approval letters.

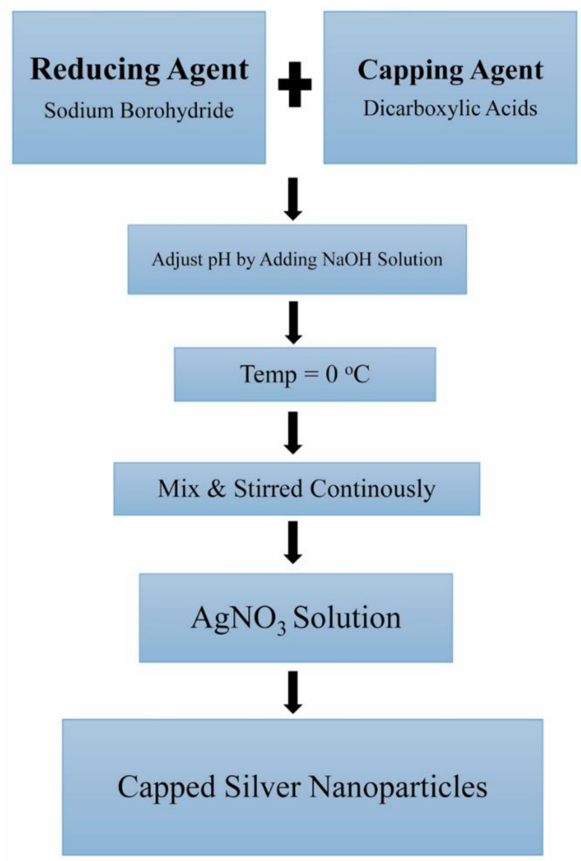

Scheme 2. The synthesis mechanism of silver nanoparticles by using C AgNPs

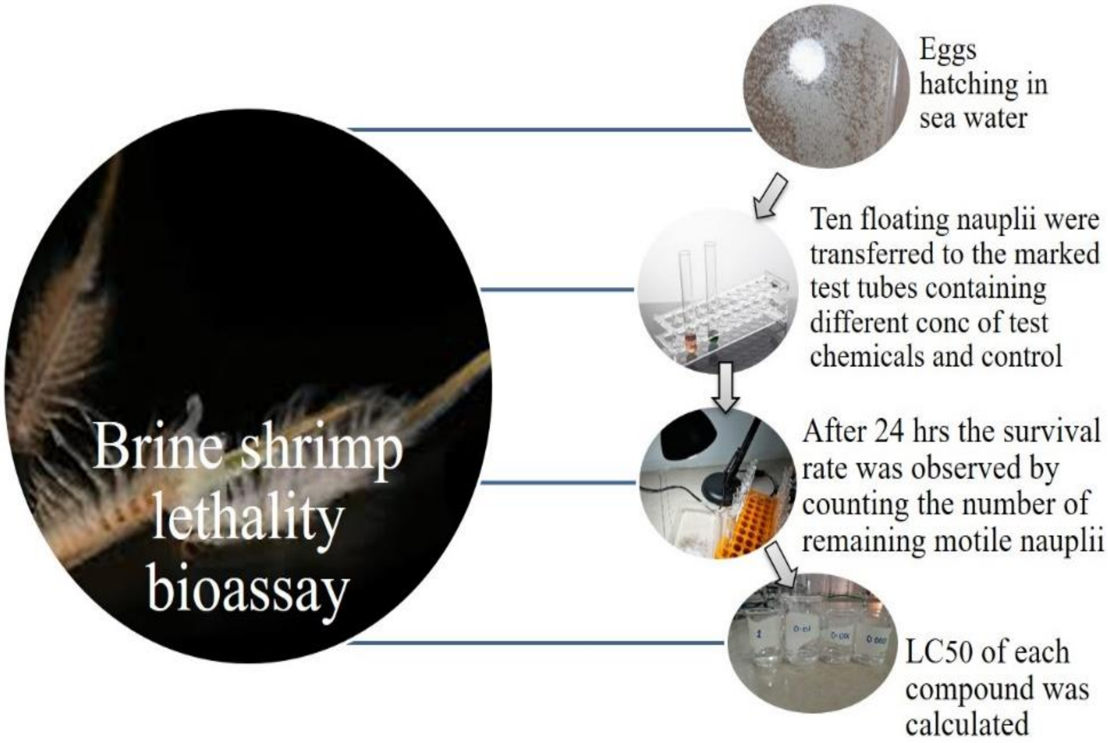

S2: Brine Shrimp Lethality bioassay

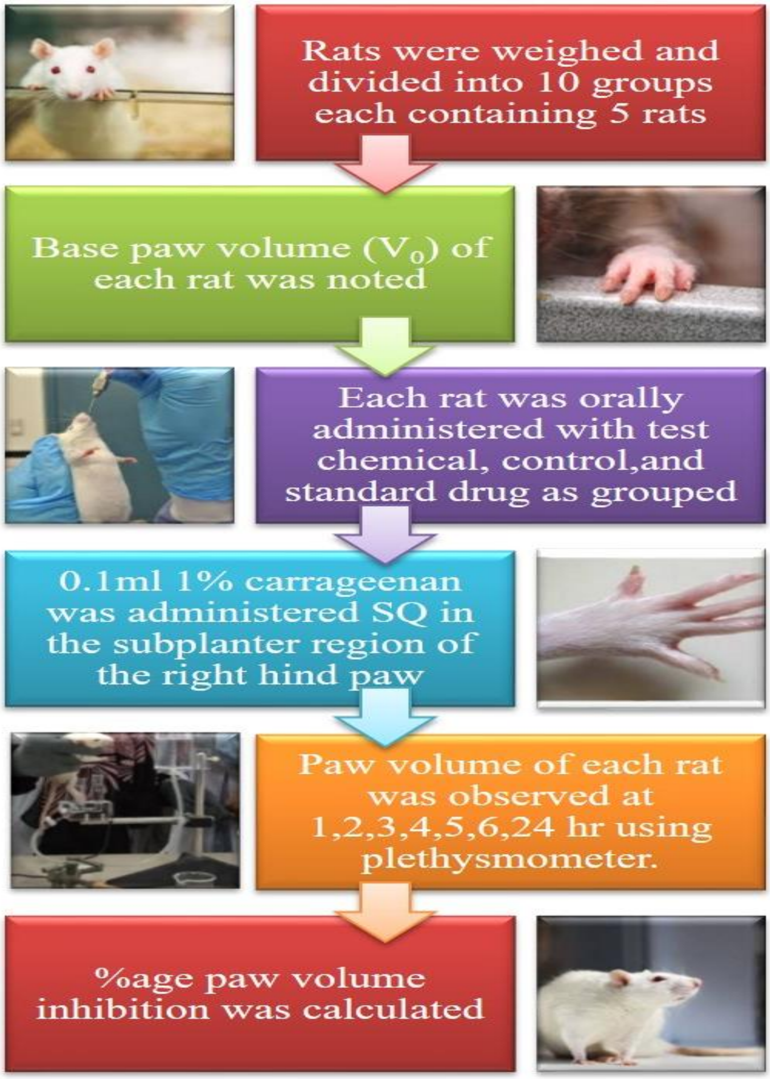

S3: Carrageenan induced paw edema method

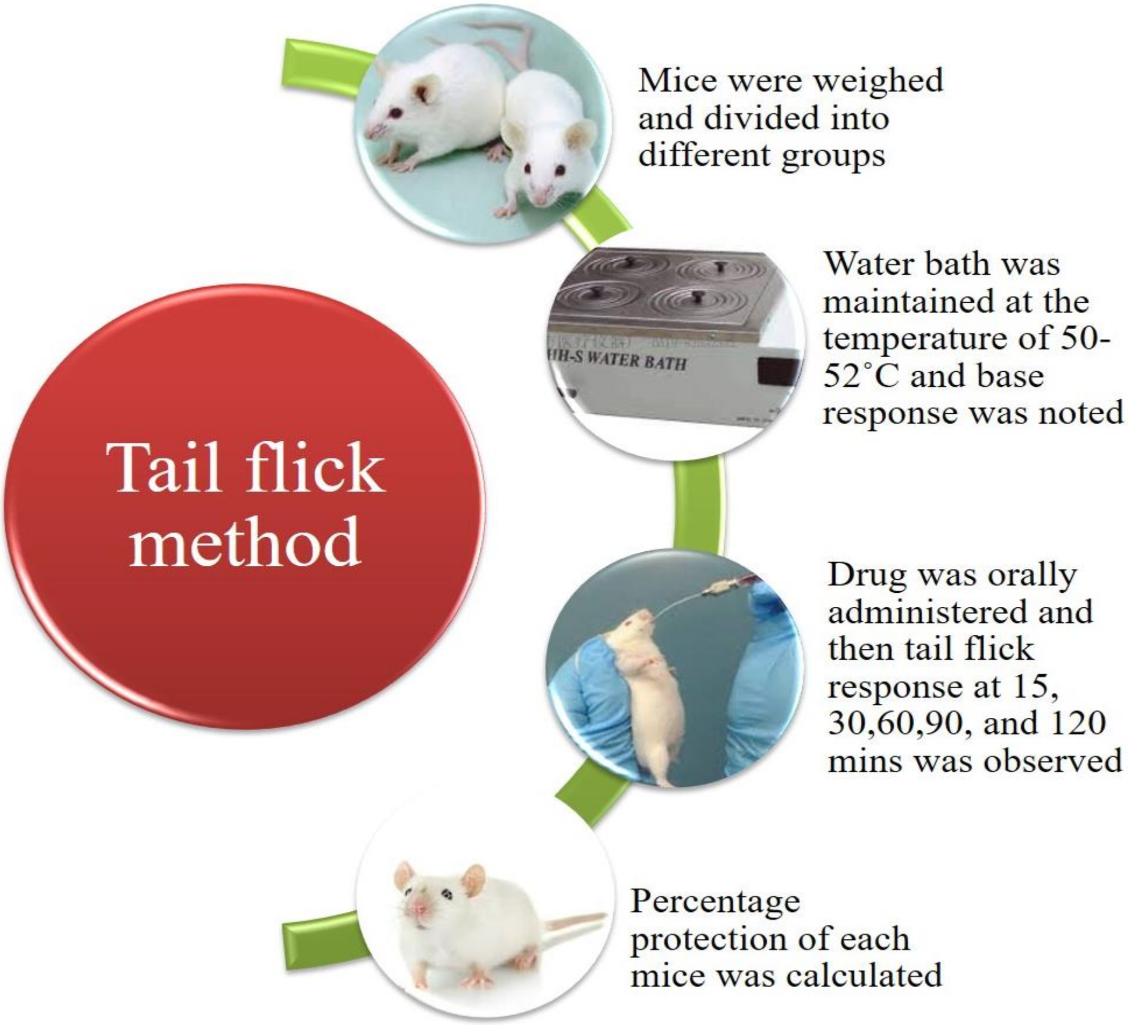

S4: Tail Flick Method

S5: LIST OF ABBREVIATIONS

˚C	Degree Celcius
µg	Microgram
µl	Microliter
µl	Microliter
µM	Micromolar
Ag	Silver
$AgNO_3$	Silver Nitrate
AgNPs	Silver Nanoparticles
AgNPs (C)	Sodium Borohydride Malonic acid capped Silver Nanoparticles
ANOVA	Analysis of variance
COX-2	Cyclooxygenase 2 enzyme
DLS	Dynamic Light Scattering
DNA	Deoxyribonucleic Acid
DPPH	2, 2 diphenyl-1-picrylhydrazyl
FT-IR	Fourier Transform Infrared Spectroscopy
g	Gram
GI	Gastrointestinal
Gram +ve	Gram-positive
Gram –ve	Gram-negative
H&E	Hematoxylin and Eosin
IC_{50}	Half maximal inhibitory concentration
IL-6	Interleukin-6
IP	Intraperitoneal

LC_{50}	Lethal Concentration 50
LD_{50}	Lethal Dose 50
M	Molar
Max	Maximum
Mg	Milligram
MIC	Minimum Effective Concentration
Min	Minimum
Ml	Milliliter
mM	Millimolar
$NaBH_4$ AgNPs	Sodium Borohydride Silver Nanoparticles
NFκB	Nuclear Factor Kappa B
Nm	Nanometer
NO	Nitric Oxide
NSAIDs	Non-Steroidal Anti-Inflammatory Drugs
OECD	Organization of Economic Co-operation and Development
PBS	Phosphate Buffer Saline
PGE2	Prostaglandin E2
pH	Potential of Hydrogen
RM ANOVA	Repeated Measure Analysis of Variance
ROS	Reactive Oxygen Species
RT-PCR	Real-Time Polymerase Chain Reaction
SD	Standard Deviation
SEM	Scanning Electron Microscope
SOD	Superoxide Dismutase
SPSS	Statistical Package for Social Sciences

Std	Standard
TAC	Total Antioxidant Capacity
TCA	Trichloroacetic Acid
TEM	Transmission Electron Microscopy
TNF-α	Tumor Necrosis Factor alpha
TSC AgNPs	Trisodium citrate Silver Nanoparticles
UV visible	Ultraviolet-visible
VS	Vincristine Sulfate

Dow University of Health Sciences
Institutional Review Board (IRB)

Ref: IRB-2698/DUHS/Approval/2022/1037 Dated: 22nd September, 2022

Ms. Tehrim Fatima
Enr # 15/2020/501
M.Phil Candidate,
Discipline of Pharmacology,
Dow College of Pharmacy
Dow University of Health Sciences.

Subject: Institutional Review Board's approval for a research proposal.

Title of Study: Evaluation of anti-inflammatory, antinociceptive and antioxidant activities of malonic acid capped silver nanoparticles

Enrolment Duration: 16-Nov-2020 to 15-Nov-2024

CRR Due Date: 21st September, 2023

Dear Ms. Tehrim Fatima,

Thank you for submitting the above mentioned study proposal. I am pleased to inform you that the IRB-DUHS has reviewed this proposal in its **185th meeting** held on **03rd September, 2022** and gives approval for a period of one year to conduct this study. You can now proceed for BASR approval.

It should be noted that thesis must be submitted and get BASR approval after successful defense on or before 15-Nov-2024. Any change in the protocol or extension in the period of study should be notified to the board for approval. Interim report on progress of study should be submitted to IRB from time to time.

Prof. Rashid Qadeer
Professor of Medicine Unit-II
Chairperson Institutional Review Board,
Civil Hospital Karachi &
Dow University of Health Sciences,
Karachi

DOW UNIVERSITY OF HEALTH SCIENCES
BOARD OF ADVANCED STUDIES & RESEARCH (BASR)

***Ref. No:** DUHS/BASR/2022/12-78*

BASR Meeting No.69
November 28, 2022

Dated: 20th Dec, 2022

SUBJECT: APPROVAL OF RESEARCH SYNOPSIS

Reference to your application for approval of research synopsis for the thesis of **Master of Philosophy (Pharmacology)**

This is to inform that your request for approval of research synopsis for the thesis of **MPhil (Pharmacology)** has been **approved** by the Board of Advanced Studies and Research (BASR), Dow University of Health Sciences w.e.f. **28-11-2022**. You will be working at Dow College of Pharmacy (DCOP), on the following research topic under the supervision of **Dr. Hina Abrar** and co-supervision **Prof. Dr. Noor Jahan and Dr. Sana Shamim.**

TOPIC

"Evaluation of Anti-Inflammatory, Antinociceptive and, Antioxidant Activities of Malonic Acid Capped Silver Nanoparticles"

You are therefore required to complete the formalities of research work and submit the thesis for **MPhil (Pharmacology)** program within maximum allowed duration as per DUHS policy.

If any forgery/fabrication/falsification/plagiarism is found in your dataset/research/thesis, as per DUHS policy you will be expelled from **MPhil (Pharmacology)** program.

Prof. Kashif Shafique
MBBS (Dow), MPH (Glasgow), PhD (Glasgow)
Secretary, Board of Advanced Studies and Research
Dow University of Health Sciences, Karachi.

Tehrim Fatima
Enr # 15/2020/501
M.Phil. (Pharmacology) Candidate
Dow College of Pharmacy (DCOP),
Dow University of Health Sciences.

Copy for information to:

- Program Director: **DCOP**
- Supervisor: **Dr. Hina Abrar**, Assistant Professor, Dept. of Pharmacology, DCOP - DUHS
- Co-Supervisor: **Prof. Dr. Noor Jahan**, Professor, Dean and Program Director of Post Graduate Studies, DCOP –DUHS.
- Co-Supervisor: **Dr. Sana Shamim**, Assistant Professor, Department of Pharmaceutical Chemistry, DCOP,

DOW UNIVERSITY OF HEALTH SCIENCES

ETHICAL REVIEW BOARD FOR ANIMAL RESEARCH & ETHICS

REF: AR.IRB-21/DUHS/Approval/2022/44

Date: July 27th, 2022

Tehrim Fatima
M.Phil Scholar
Dow College of Pharmacy
Dow University of Health Sciences

Subject: Ethical Review Board for Animal Research & Ethics Approval for Research Proposal

Title of Study: Evaluation of anti-inflammatory, antinociceptive and antioxidant activities of malonic acid capped silver nanoparticles

Tehrim Fatima

Thank you for submitting the above-mentioned study proposal. I am pleased to inform you that ERB for Animal Research & Ethics, DUHS has reviewed this research proposal in its 25th meeting held on July 19th, 2022 and gives approval to conduct procedure incorporating animals as mentioned in study design for a period of one year.

Any changes in the animal-based procedure/protocol or extension in the duration of study should be notified to board for the approval. Progress report and concluding reports should be submitted to committee.

Prof Dr. Zeba Haque
Professor of Biochemistry
Dow International Medical College
Chairperson,
Ethical Review Board for Animal Research & Ethics
Dow University of Health Sciences
Karachi

DOW UNIVERSITY OF HEALTH SCIENCES
SCIENTIFIC COMMITTEE

Ref. No: DUHS/SC/2022/- 172
Date: 21st May, 2022

Ms. Tehrim Fatima
Enr # 15/2020/501
M.Phil Candidate,
Discipline of Pharmacology,
Dow College of Pharmacy,
Dow University of Health Sciences.

Subject: Approval of M.Phil (Pharmacology) Candidate's Synopsis by Scientific Committee

Enrolment Duration: 16-Nov-2020 to 15-Nov-2024

Dear Ms. Tehrim Fatima,

I would like to inform you that your synopsis titled **"Evaluation of anti-inflammatory, antinociceptive and, antioxidant activities of malonic acid capped silver nanoparticles"** was presented in 118th Scientific Committee Meeting for review held on April 29, 2022. The synopsis was revised and resubmitted with the marked corrections. Therefore, it has been approved by the Scientific Committee of Dow University of Health Sciences for M.Phil (Pharmacology) research. You can now process for further approvals from IRB and BASR.

It should be noted that thesis must be submitted and get BASR approval after successful defense on or before 15th November 2024.

Thanks and regards,

Yours Sincerely,

Prof. Kashif Shafique
MBBS (Dow), MPH (Glasgow), PhD (Glasgow)
Secretary, Scientific Committee,
Professor (Public Health) & Principal, School of Public Health,
Director, ORIC,
Dow University of Health Sciences, Karachi, Pakistan.

Copy to:

1. Record file
2. Secretary, BASR: Board of Advanced Studies and Research.
3. Program Director & Co-Supervisor: Prof. Dr. Noor Jahan, Dean, Dept. of Pharmacology, DCOP, DUHS.
4. Supervisor: Dr. Hina Abrar, Asst. Prof., Dept. of Pharmacology, DCOP, DUHS.
5. Co-Supervisor: Dr. Sana Shamim, Asst. Prof., Dept. of Pharmaceutical Chemistry, DCOP, DUHS.
